# Role of PD-1/PD-L1 signaling axis in oncogenesis and its targeting by bioactive natural compounds for cancer immunotherapy

**DOI:** 10.1186/s40779-024-00586-9

**Published:** 2024-12-18

**Authors:** Yogesh Godiyal, Drishti Maheshwari, Hiroaki Taniguchi, Shweta S. Zinzuwadia, Yanelys Morera-Díaz, Devesh Tewari, Anupam Bishayee

**Affiliations:** 1 Department of Pharmacognosy and Phytochemistry, School of Pharmaceutical Sciences, Delhi Pharmaceutical Sciences and Research University, New Delhi, 110017 India; 2 Department of Experimental Embryology, Institute of Genetics and Animal Biotechnology of the Polish Academy of Sciences, Jastrzebiec, 05-552 Magdalenka, Poland; 3 African Genome Center, Mohammed VI Polytechnic University, Hay Moulay Rachid, 43150 Ben Guerir, Morocco; 4 Department of Pharmacology, College of Osteopathic Medicine, Lake Erie College of Osteopathic Medicine, Bradenton, FL 34211 USA; 5 Clinical Investigation and Biomedical Research Directions, Center for Genetic Engineering and Biotechnology, 11600 Havana, Cuba

**Keywords:** Cancer, PD-1/PD-L1, Crosstalk, Natural compounds, Therapeutic targets

## Abstract

Cancer is a global health problem and one of the leading causes of mortality. Immune checkpoint inhibitors have revolutionized the field of oncology, emerging as a powerful treatment strategy. A key pathway that has garnered considerable attention is programmed cell death-1 (PD-1)/programmed cell death ligand-1 (PD-L1). The interaction between PD-L1 expressed on tumor cells and PD-1 reduces the innate immune response and thus compromises the capability of the body’s immune system. Furthermore, it controls the phenotype and functionality of innate and adaptive immune components. A range of monoclonal antibodies, including avelumab, atezolizumab, camrelizumab, dostarlimab, durvalumab, sinitilimab, toripalimab, and zimberelimab, have been developed for targeting the interaction between PD-1 and PD-L1. These agents can induce a broad spectrum of autoimmune-like complications that may affect any organ system. Recent studies have focused on the effect of various natural compounds that inhibit immune checkpoints. This could contribute to the existing arsenal of anticancer drugs. Several bioactive natural agents have been shown to affect the PD-1/PD-L1 signaling axis, promoting tumor cell apoptosis, influencing cell proliferation, and eventually leading to tumor cell death and inhibiting cancer progression. However, there is a substantial knowledge gap regarding the role of different natural compounds targeting PD-1 in the context of cancer. Hence, this review aims to provide a common connection between PD-1/PD-L1 blockade and the anticancer effects of distinct natural molecules. Moreover, the primary focus will be on the underlying mechanism of action as well as the clinical efficacy of bioactive molecules. Current challenges along with the scope of future research directions targeting PD-1/PD-L1 interactions through natural substances are also discussed.

## Background

Multiplication is the fundamental and inherent characteristic of every cell, leading to the generation of new life. Each cell possesses the ability to multiplicate, resulting in the production of new daughter cells. Cancer cells, however, form colonies that are more uncontrollable and proliferate more frequently than normal cells, exhibiting invasive and metastatic properties [1]. Recent statistics state that in 2024, there will be an estimated 611,720 cancer-related deaths and 2,001,140 new cancer cases projected in the United States [2]. Cancer mortality rates decreased until 2021, saving almost 4 million lives since 1991 as a result of decreased smoking rates, early cancer identification for certain types of cancer, and enhanced adjuvant and metastatic cancer treatment choices [2]. The cancer burden is expected to rise to approximately 28 million new cases and 16 million deaths by 2040 [3].

Although increasing technological advancements have led to a decline in cancer mortality rates, cancer is still the second leading cause of death globally [1]. In various cases, the uncontrolled multiplication, aggressiveness, and immortality of cancer cells result from genetic variations, inheritance patterns, or exposure to specific carcinogenic agents [1]. Several factors, such as increasing life expectancy, developmental status, and various reproductive and non-reproductive aspects, influence the disease burden [4, 5]. It is estimated that until 2040, cancer management in the older population could be a complex issue with high comorbidity rates, declining body functions, limited life expectancy, and increased vulnerability among this demographic [6].

While various signaling pathways require growth factors for activation, cancerous cells can be activated even in the absence of these factors due to mutations [1]. Role of specific pathways, such as mitogen-activated protein kinase (MAPK), phosphatidylinositol 3-kinase/protein kinase B (PI3K/Akt) along with its negative regulator phosphatase and TENsin homolog (PTEN), Hedgehog (Hh), and the Hippo pathways involved in cancer cell proliferation and survival have been elucidated by numerous researchers [7–11]. Similarly, many additional pathways contribute to cancer progression, including activator protein 1 [12], the Notch signaling pathway [13, 14], transforming growth factor-β (TGF-β) [15], and the wingless/integrated** (**Wnt)/β-catenin pathway [16].

One important and relatively less explored pathway is the programmed cell death protein 1 (PD-1)/programmed cell death ligand (PD-L1) pathway [7]. PD-1 is a surface receptor expressed by T cells and consists of 288 amino acids, classifying it as a type I membrane protein. As a T cell regulator, PD-1 belongs to the expanded cluster of differentiation 28 (CD28)/cytotoxic T lymphocyte-associated protein 4 (CTLA-4) family [17]. The structure of this protein comprises an intracellular tail, a transmembrane segment, and an external immunoglobulin variable domain. Within the intracellular tail, there are two phosphorylation sites, one in an immunoreceptor tyrosine-based switch motif and the other in an immunoreceptor tyrosine-based inhibitory motif. This suggests that PD-1 inhibits T cell receptor (TCR) signals [17, 18]. The interaction between PD-1 and its ligands, PD-L1 and programmed cell death ligand 2 (PD-L2), on tumor cells leads to the downregulation of T cell response in the tumor microenvironment. Concurrently, CTLA-4 mainly expressed in T cells, competes with the CD28 receptor for binding to these ligands, resulting in the downregulation of immune responses [19].

Drugs targeting the PD-1/PD-L1 interaction and facilitating therapies for cancer prevention have garnered peculiar attention over the past decade. With the approval of pembrolizumab [20, 21] and nivolumab in 2014 [22, 23], followed by the acceptance of avelumab [24] and atezolizumab [25], dostarlimab is the recently approved drug for cancer immunotherapy [26, 27]. A recent clinical experiment conducted on colorectal cancer patients observed that the monoclonal antibody (mAb), dostarlimab, led to the complete eradication of cancer cells without any residual cells left behind or potential for reoccurrence [28]. This effect was attributed to the inhibition of PD-1 and the blockade of interaction between PD-L1 and PD-L2, thus activating T cells and enhancing overall immunity. These findings highlight the high efficacy of this drug in treating cancer patients [28]. Therefore, PD-1/PD-L1 is a potential therapeutic target for clinical management of cancer.

Four primary categories of immunotherapeutic resistance mechanisms associated with the PD-1/PD-L1 pathway have been identified, which are microbiota modifications, metabolic abnormalities, epigenetic alterations, and immunosuppression [29]. Primary and acquired resistance to PD-1/PD-L1 inhibition can arise from a variety of pathways, some of which may be complex and exhibit overlap among patients. Improved patient outcomes are anticipated when new approaches to prevent or reverse therapeutic resistance are developed, particularly as the mechanisms underlying resistance to PD-1/PD-L1 blockade continue to be more characterized [30]. A detailed overview of the resistance mechanisms of PD-1/PD-L1 blockade has been published elsewhere [30, 31]. It is imperative to understand these resistance mechanisms and the development of new drugs either synthetic or natural compound-based drug delivery. Addressing these issues could prove highly beneficial in overcoming challenges associated with resistance.

Natural remedies have been utilized historically and in folklore to address a wide range of ailments since prehistoric times. An enormous variety of bioactive secondary metabolites from both terrestrial and marine sources have been identified through natural product chemistry techniques. Numerous of these natural compounds are currently being explored as potential therapeutic agents [32]. Additionally, compared to conventional chemotherapy medications, phytochemicals offer a broader therapeutic index. Therefore, patients with cancer may benefit from the use of phytochemicals either alone or in conjunction with approved chemotherapies [33].

While the PD-1/PD-L1 pathway has gained significant attention in immunotherapy, the integration of natural bioactive compounds introduces a novel dimension to this field. Plant secondary metabolites or phytochemicals are naturally occurring compounds, many of which have been implicated in cancer treatment. A previous compilation review mentioned 247 natural and derived products indicated or approved for their anticancer potential from January 1981 to September 2019 [34]. Preceding studies have highlighted the impact of various phytochemicals in targeting the PD-1/PD-L1 axis across several carcinomas. This is achieved through the expression of intracellular molecules, posttranslational modifications, and regulation of signaling pathways, making them effective modalities for anticancer therapy [35–37]. Natural compounds represent a vital source of lead molecules, encompassing a combination of both natural agents and their structurally modified analogs. When integrated with immune checkpoint inhibitors (ICIs), these compounds are expected to provide safer and more effective options for anticancer treatment [36].

Previous reviews have effectively underscored the role of PD-1/PD-L1 targeting, although only a limited number of available phytochemical studies have been conducted regarding the prevention of tumor growth [37–39]. Research focusing on the PD-1/PD-L1 interaction in the context of tumor growth inhibition remains scarce [37, 40]. It is crucial to deepen our understanding of the possible mechanisms underlying anticancer therapies, particularly those involving bioactive natural compounds, within cancer treatments that target the PD-1/PD-L1 interaction.

Following a thorough review of the relevant literature, it has been found that there is no comprehensive study available that emphasizes the impact of a diverse array of natural compounds against PD-1/PD-L1. Our review focuses on a large number of studies involving natural agents and provides an in-depth analysis of published results. Therefore, this is the first attempt where a cutting-edge approach is adopted that harnesses the therapeutic potential of bioactive natural compounds to modulate the PD-1/PD-L1 signaling pathway for effective cancer treatment. Additionally, this review comprehensively highlights a range of clinical studies performed or are currently ongoing with multiple natural substances, while discussing the shortcomings and challenges associated with using these agents for cancer therapy in a clinical setting. By investigating the intricate interactions between bioactive natural compounds and the PD-1/PD-L1 pathway, this work bridges traditional medicine, with a focus on natural compounds, and modern immunotherapy, offering a fresh perspective on cancer therapy. This innovative approach not only leverages the extensive reservoir of natural compounds with established pharmacological activities but also capitalizes on the potential synergies between these compounds and immunomodulatory pathways. Here, this paper aims to provide a comprehensive overview of possible targeting strategies of PD-1 and its respective ligand by a range of natural compounds derived from plants, marine, and microbial sources along with detailed mechanisms of action.

## Role of PD-1 in cancer

Various regulatory mechanisms are involved in the control of tumor cell proliferation and immune tolerance in tissues. Immune checkpoint molecules are one such mechanism through which tumor cells evade detection by the immune system [19]. PD-1 and its ligands are expressed in various cell types of the innate and adaptive immune system, and their expression is regulated at both transcriptional and posttranslational levels [41]. The upregulation of PD-1 is a natural phenomenon that occurs following T cell activation, resulting in beneficial effects in protecting peripheral tolerance and preventing autoimmune diseases. Tumors escape host immune surveillance by expressing PD-L1, which negatively regulates immune responses through its interaction with PD-1, thereby leading to T cell apoptosis [42, 43].

The binding of PD-1 on T cells to PD-L1 on tumor cells triggers inhibitory signaling that attenuates T cell responses. The inhibitory signals are blocked by antibodies against PD-1 and its ligands, whereas the role of PD-L2 remains controversial due to its dual costimulatory and coinhibitory functions [41]. By inhibiting lymphocyte growth and activation, the interaction between PD-1 and PD-L1 transmits inhibitory signals within cells, thus reducing immunological potential. As a result, tumor cells frequently “hijack” this axis to promote their survival and proliferation [44, 45]. Immune checkpoint proteins act as switches for immune activity, controlling the activation or inactivation of immune responses. For example, PD-1 and its ligand PD-L1 negatively regulate the balance and functionality of T cell immune responses. T cells are capable of recognizing and eliminating harmful or infected cells, including cancerous ones [46]. The presence of PD-1 in T cells is common, while cancer cells frequently express PD-L1. The binding of PD-1 to PD-L1 may lead to reduced T cell activity and antitumor immunity by activating an inhibitory signal [47].

### PD-1/PD-L1 signaling in various cancer-related molecules and pathways

Certain tumors exhibit adaptive immune resistance by expressing PD-L1 reactively and adaptively in response to inflammatory cytokines, including tumor necrosis factor-α (TNF-α), thus preventing immune-mediated assaults [48]. The primary mechanism of anti-PD-1 or anti-PD-L1 therapies is to prevent the interaction between cell surface-expressed PD-L1 and PD-1^+^ antitumor T cells, resulting in decreased T cell death and increased memory formation [49]. Various pathways, including MAPK, Janus kinase (JAK)/signal transducer and activator of transcription (STAT), nuclear factor-κB (NF-κB), Wnt, and Hh signaling pathways have been shown to affect the signaling of PD-1/PD-L1 in tumor cells [50].

#### Interferon-γ (IFN-γ)

IFN-γ receptor belongs to the class 2 cytokine family and is expressed by all cells except erythrocytes. It consists of two subunits, namely interferon-γ receptor 1 and interferon-γ receptor 2 [50]. Activation of IFN-γ leads to increased cytokine synthesis, enhanced phagocytosis, and cytotoxic activity. Tumor cells can evade immune responses through the upregulation of membrane-bound inhibitory molecules [51]. IFN-γ mediates upregulation of PD-L1 and facilitates its binding to PD-1 on T cells, resulting in a suppressed immune response [51]. IFN-γ is mainly produced by natural killer (NK) and natural killer T (NKT) cells, which subsequently activate the JAK/STAT signaling pathway [51]. This cytokine performs multiple functions, including antigen processing, regulation of B cell proliferation, generation of T helper type 1 (Th1) CD4^+^ helper T cells, downregulation of protumorigenic actions by regulatory T cells (Tregs), and modulation of CD8^+^ T cell proliferation [51]. Flow cytometry analysis revealed that the presence of IFN-γ in the glioma microenvironment upregulates the expression of PD-L1 while contributing to T cell dysfunction and apoptosis coupled with immune suppression [52].

In vitro experiments conducted on various oral squamous cancer cell lines showed that IFN-γ increases the expression of PD-L1 along with the phosphorylation of the downstream molecule STAT1 [53]. Experiments on melanoma cell lines have also indicated significant upregulation of PD-L1 expression with the activation of several transcriptional genes, specifically *JAK2*, *STAT1*, *STAT2*, *STAT3*, *IRF1* (interferon regulatory factor 1), and *IRF9*, in response to IFN-γ stimulation [54]. In vitro tests using human hepatocyte cells further suggest that IFN-γ induces PD-L1 expression in a concentration-dependent manner, leading to increased levels of *PD-L1* mRNA [55]. Flow cytometry analysis involving ovarian cancer patients indicates that IFN-γ can induce PD-L1 expression in previously negative cases following incubation with IFN-γ, highlighting its induction potential [56]. In vivo mouse models demonstrated a decline in tumor progression upon knockdown of *PD-L1*, thus exhibiting a strong correlation between IFN-γ and PD-L1 [56].

#### Interleukin-6 (IL-6)

IL-6, a glycopeptide, is a member of the proinflammatory cytokine family. It plays a crucial role in various biological functions, including inflammation, proliferation, and differentiation, along with effects on tumor growth, apoptosis, angiogenesis, and metastasis [57]. A study involving colorectal cancer patients indicates that IL-6 induces immunosuppression, upregulates PD-L1 levels, and promotes tumor progression. Furthermore, it was observed that anti-IL-6 therapy could reverse the resistance to anti-PD-L1 treatment in colorectal cancer cells [58]. Another investigation on ovarian cancer patients suggests that elevated IL-6 levels are associated with shorter survival times, and reveals a negative relationship between CD45^+^CD14^+^PD-L1^+^ cells and IL-6 levels [59]. The upregulation of IL-6 is further linked to increased PD-L1 expression and poor prognosis in hepatic tumors mediated through the proteasome pathway [60]. Additionally, in vivo experiments demonstrated that treatment with an IL-6 antibody decreases PD-L1 expression in tumor regions by modulating its glycosylation while enhancing JAK1/nonglycosylated PD-L1 association, JAK1-driven Tyr phosphorylation, and STAT3 recruitment [60]. The study on non-small cell lung cancer (NSCLC) cells showed a positive correlation between IL-6 expression and PD-L1 expression alongside the infiltration of myeloid-derived suppressor cells (MDSCs), M2 macrophages, and Tregs, highlighting its critical role in the suppression of immune response [61]. Overexpression of IL-6 in thyroid cancer cells is related to an increase in PD-L1 protein levels, predominantly mediated through M2 macrophages [62]. Recent studies indicate heightened myeloid infiltration corresponding to higher tumor-associated IL-6 expression in glioblastoma patients [63, 64]. Suppressing IL-6 levels results in decreased PD-L1 expression within the tumor microenvironment, leading to diminished tumor growth and enhanced survival outcomes [64].

#### MAPK pathway

The MAPK signaling pathway is a vital regulator of cellular processes such as proliferation, differentiation, invasion, metastasis, and phosphorylation-mediated death [50]. Research on breast cancer cell lines demonstrated that the inhibition of MAPK pathway suppresses PD-L1 expression. Interestingly, the interaction between PD-1 and PD-L1 leads to enhanced activation of the MAPK/extracellular signal-regulated kinase (ERK) pathway [65]. Targeted therapies that inhibit MAPK have shown increased tumoral infiltration and improved tumoral control when combined with anti-PD-1 therapy [66]. In lung cancer cell lines, selumetinib, a MAPK inhibitor, was found to decrease PD-L1 levels even at low concentrations, suggesting a reduction in PD-L1 protein and membrane expression while also destabilizing *PD-L1* mRNA [67]. Furthermore, activation of the MAPK pathway suppresses major histocompatibility complex (MHC)-I and MHC-II expression; however, synergistic inhibition of mitogen-activated extracellular signal-regulated kinase (MEK) alongside PD-L1 leads to the downregulation of immunosuppressive factors and upregulation of MHC-I and MHC-II expression in triple-negative breast cancer (TNBC) cells, effectively reversing these actions [68]. Both in vitro and in vivo experiments conducted on pancreatic cancer (PC) cell lines have further concluded that MAPK signaling serves as a key regulator of PD-L1 expression in the tumor microenvironment [69]. Additionally, MAPK activation promotes increased levels of PD-L1 through upregulation of c-Jun and STAT3. Knockdown of these kinases sufficiently suppressed PD-L1 expression while demonstrating combinatorial synergistic effects compared to inhibiting individual kinases alone [70].

#### PI3K/Akt/mammalian target of rapamycin (mTOR) pathway

PI3K/Akt/mTOR pathway is one of the most utilized and activated signaling cascades in various human cancers. It is extensively deregulated due to oncogenic mutation or signal amplification, resulting in a loss of tumor cell suppressive functions [71]. Hyperactivation of the PI3K/Akt pathway has been associated with resistance against epidermal growth factor receptor (EGFR) inhibitors, as well as enhanced cancer cell invasion and metastasis [72, 73]. Studies indicated a positive correlation between suppression of the human epidermal growth factor receptor 2 (HER2)/PI3K/Akt pathway and PD-L1 expression [74, 75], with heat shock protein 90 (HSP90) regulating tumor expression in certain cancers [76]. Further findings demonstrated that *HSP90* knockdown suppressed HER2 expression and destabilized the PI3K/Akt pathway, resulting in a downregulation of PD-L1 and the inhibition of cancer cell proliferation and migration [77]. In myeloid leukemia, PD-L1 expression was mediated through the P13K/Akt signaling pathway and interactions with the extracellular matrix receptors, indicating a close relationship with PD-L1 levels [78]. Research on nasopharyngeal cancer cells has shown that overexpression of PD-L1 in vitro enhances cellular migration and invasion via alterations in epithelial-mesenchymal transition (EMT)-like cellular markers alongside activation of the PI3K/Akt signaling pathway. Suppression of PD-L1 expression reverses these EMT-like molecular changes while further reducing migration and invasion by inhibiting the PI3K/Akt signal pathway [79]. In vivo experiments suggest that inhibition of the PI3K/Akt/mTOR axis induces apoptosis mediated by PD-1 while also inhibiting CD4^+^ and CD8^+^ T cell proliferation [80]. Experiments conducted using Lewis lung carcinoma (LLC) cells demonstrate the activation of PI3K/Akt/mTOR pathway and its suppression by anti-PD-1 therapy, could result in activation of autophagy, suppression of angiogenesis and inhibition of tumor growth [81]. In lung adenocarcinoma cells, inhibition of PI3K leads to a reduction in *PD-L1* mRNA and protein expression [82].

#### JAK/STAT pathway

The JAK/STAT pathway serves as a major component of the cytokine-mediated signal transduction network and is involved in various biological processes, including proliferation, differentiation, organ development, and immune homeostasis. Dysregulation in the homeostasis of the JAK/STAT signaling pathway is considered to be a contributing factor in autoimmune disorders and a variety of tumors [83, 84]. A study conducted on PC cell lines has shown that increased levels of PD-L1 are reduced at mRNA and protein levels by the induction of JAK2 inhibitors and enhanced phosphorylation of STAT1, thereby demonstrating the regulatory role of JAK/STAT pathway on PD-L1 expression [85]. Additionally, cAMP induces the activation of PD-L1 through the secretion of ILs and subsequent activation of the JAK/STAT signaling pathway [86]. In vitro experiments have suggested a positive correlation between IL-4 induced gene 1 (*IL4I1*) expression and PD-L1 levels. This relationship was further confirmed through a bioinformatics analysis, showing that *IL4I1* silencing suppresses PD-L1 expression while reducing T cell toxicity through the JAK/STAT signaling cascade [87]. In a tumor microenvironment, the transphosphorylation of JAK1 [88], JAK2, and STAT1 led to the upregulation of PD-L1 expression [89, 90]. Furthermore, the inhibition of JAK2 resulted in a decrease in PD-L1 expression [91]. In breast cancer cell lines, it was observed that combined inactivation of STAT1 and STAT3 signaling pathways results in significant downregulation of PD-L1 expression compared to individual inhibition [92]. Cytochrome B561 family member D2, an antioxidant protein, facilitates the expression of immunosuppressive genes via the activation of STAT3 and enhanced PD-L1 expression, which is also confirmed by database analysis [93].

#### NF-κB

NF-κB belongs to a family of dimeric transcription factors that play crucial roles in numerous cellular functions. Dysregulation of NF-κB is associated with a range of conditions, including tumors, inflammation, and immune disorders [94, 95]. Two primary pathways, the anonical NF-κB essential modulator (NEMO)-dependent pathway and the noncanonical NEMO-independent pathway, are responsible for the regulation of the NF-κB network [94]. These are mediated by inhibitors of nuclear factor-κB (IκB) kinase (IKK) α and IKKβ, along with some other regulators [96, 97]. Experimental evidence has established a link between IKKβ expression and various factors, such as cyclooxygenase-2, matrix metalloproteinase-9 (MMP-9), and macrophage inflammatory protein 2. These factors contribute to the growth of tumors and are further amplified by NF-κB activation [98]. In Hodgkin’s lymphoma, the activation of PD-L1 and PD-L2 is mediated via the NF-κB signaling pathway, which promotes immune escape by activating transcription factors [99]. Studies have demonstrated that IFN-γ inducible protein 16 enhances the stimulation of interferon genes, specifically TRAF family member-associated NF-κB activator binding kinase 1, resulting in downstream activation of the NF-κB pathway. This process emphasizes its effect on PD-L1 expression while facilitating cervical cancer progression [100, 101].

#### microRNAs (miRNAs)

miRNAs are short non-coding RNAs that play a vital role in various regulatory pathways, gene activation, and the regulation of cellular activities, including cell proliferation, cell cycle regulation, apoptosis, invasion, and metastasis [102]. The significance of several miRNAs, such as miR-197-5p, miR-93-5p, miR-378a-3p, and miR-98-5p, has been highlighted in previous review [103]. Dysregulation of miRNA expression can lead to protumor activity or underexpression that activates tumor-suppressing genes. This dysregulation may result from genetic loss, epigenetic modifications, widespread transcriptional repression, or defective biogenesis [103]. The regulation of PD-L1 expression by miR-200 is observed, along with CD8^+^ cell infiltration and elevated EMT scores in mesenchymal lung cancer cells [103]. In A549 cell line, overexpression of miR-155-5p results in the downregulation of *PD-L1* expression at protein and mRNA levels via two binding sites located in the 3’-UTR of PD-L1 [104]. Previous studies have described the interaction between miR 105-5p and PD-L1 expression [105], with specific binding occurring at the F522 site within the 3’UTR of the PD-L1 [106, 107]. Furthermore, in vitro analyses confirmed that transfection with miR-105-5p into HEK293T and A549 cells resulted in PD-L1 downregulation while promoting immune surveillance via T cell activation [108]. Breast cancer cells also exhibit potential for immune escape under endoplasmic reticulum stress conditions, leading to miR-27a-3p production. This further upregulates PD-L1 expression and enhances immune evasion in macrophages [109]. It has been indicated that the regulation of PD-L1 expression occurs through the binding of miR-34 to its 3’UTR [110], mediated by p53. A direct interaction between miR-33a and the 3’UTR of PD-1 and PD-L1 was also observed, resulting in downregulation of their expression levels [111]. Elevated levels of miR-33a suggest a potential regulatory role in PD-1/PD-L1 [111]. In vivo experiments on malignant pleural mesothelioma cells further underscore the regulation of PD-L1 through miR-320a, targeting the 3’UTR [112].

#### Long non-coding RNAs (lncRNAs)

LncRNAs are a class of RNA molecules characterized by transcripts exceeding 200 nucleotides in length and lacking protein-coding ability. LncRNAs have been identified to play important roles in a host of regulatory mechanisms, such as tumor growth and proliferation [113–116]. Numerous lncRNAs have been implicated and highlighted in multiple types of cancer. Previous research has demonstrated the relationship between small nucleolar RNA host gene (SNHG) 14 and cancer proliferation [117]. Experimental evidence indicates elevated expression of SNHG14 in lymphoma cell lines is positively correlated with zinc finger E‐box binding homeobox 1 (ZEB1). Silencing of SNHG14 reduced mRNA expression of both *ZEB1* and *PD-L1* [118].

Another lncRNA, SNHG20, has been indicated in esophageal squamous cell carcinoma. In vitro experiments suggest that upregulation of SNHG20 expression is associated with poor prognosis and enhanced metastasis. Furthermore, inhibition of SNHG20 leads to downregulation of ataxia telangiectasia mutated phosphorylated ataxia telangiectasia-mutated kinase (p‐ATM), p‐JAK1/2, and PD-L1, thereby indicating its control mechanism [119]. Dysregulation of miR-155 host gene (MIR155HG), which encodes lncRNA-155 [120], plays a significant role in tumor progression, invasion, and metastasis, and there is a strong correlation between MIR155HG and PD-L1 [121]. Actin filament-associated protein 1 antisense RNA1 (AFAP1-AS1) is a recently identified lncRNA that is highly expressed in tumors and promotes proliferation, chemotherapy resistance, and cancer cell invasion [116, 122]. The elevated presence of AFAP1-AS1 in nasopharyngeal carcinoma is positively correlated with PD-1 based on Gene Expression Omnibus database analysis [114, 123]. It has been predicted that the high incidence of AFAP1-AS1 is related to metastasis and poor prognosis in nasopharyngeal carcinoma [114].

Urothelial carcinoma associated 1 (UCA1), another lncRNA initially identified in human bladder carcinoma, has been implicated in various other tumors [124]. A study conducted on gastric cancer cells indicates that increased UCA1 expression is correlated with poor prognosis and reduced survival rates. UCA1 overexpression enhances the motility of tumor cells, leading to downregulation of antitumor miRNAs such as miR-26a and miR-26b, while upregulation of PD-L1 levels. Conversely, knockout of UCA1 results in reduced migration of gastric cancer cells [125].

**Fig. 1:**
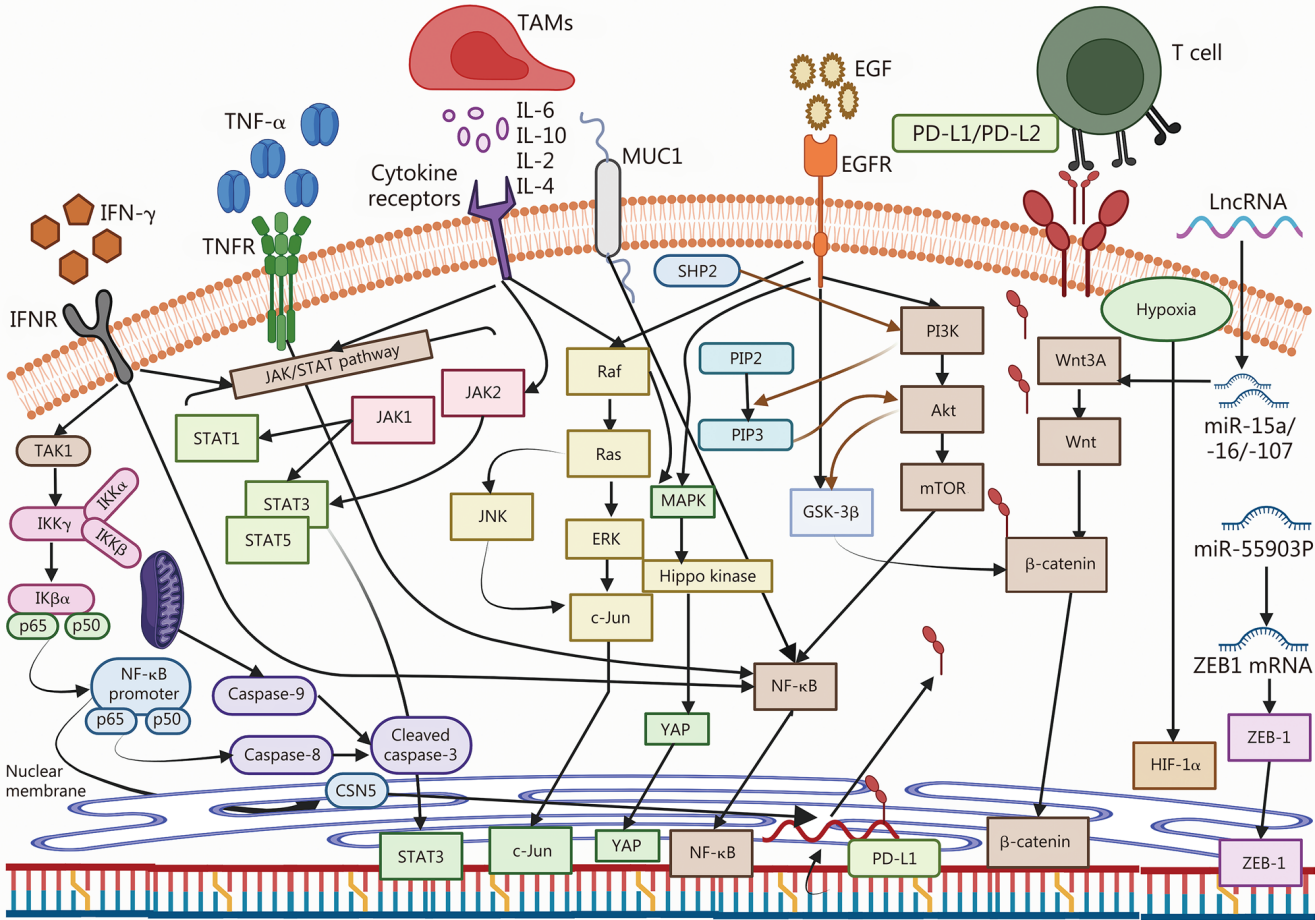
Schematic diagram of proposed signaling pathway representing PD-1, PD-L1, and their interactions. PD-L1 is an immune checkpoint protein, highly expressed in part of tumor cells, and promotes tumor cell escape from T cells. PD-1 expressed on T-lymphocytes can bind to PD-L1 and inhibit T cell proliferation and activity. IFN-γ derived from tumor-infiltrating immune cells induces transcription of PD-L1 in tumor cells via the JAK/STAT signaling pathway along with NF-κB. MAPK signaling further increased the stability of *PD-L1* mRNA, thus higher mRNA and protein levels, leading to increased PD-L1 membrane expression. IL-6 binds to IL-6R, resulting in homodimerization of the signal-transducing receptor subunit and activating the intracellular signaling cascades of JAK, STAT3, MAPK, and PI3K. Pathways converging with c-Jun include MAPK and JNK and their reactivation leads to c-Jun and STAT3 dependent enhancement of PD-L1 expression. YAP1 translocates to the nucleus interacting with transcription factors involved in the transcription of genes associated with cell growth and proliferation. Increased kinase activity of EGFR leads to hyperactivation of downstream signaling pathways including MAPK, PI3K/Akt/mTOR, and IL-6/JAK/STAT3 promoting tumorigenesis. *PD-L1* mRNA is translated into PD-L1 protein, which is transported to the cell membrane. Akt protein kinase B, CSN5 constitutive photomorphogenic-9 signalosome 5, EGF epidermal growth factor, EGFR epidermal growth factor receptor, ERK extracellular signal-related kinases, GSK-3β glycogen synthase kinase-3β, HIF-1α hypoxia inducible factor-1α, IFN-γ interferon-γ, IFNR interferon receptor, IKKα inhibitor of nuclear factor-κB kinase α, IKKβ inhibitor of nuclear factor-κB kinase β, IKKγ inhibitor of nuclear factor-κB kinase γ, IL interleukin, JAK Janus kinase, STAT signal transducers and activators of transcription, JNK c-Jun N-terminal kinase, LncRNA long non-coding RNA, MAPK mitogen-activated protein kinase, miR microRNA, mTOR mammalian target of rapamycin, MUC1 mucin 1, NF-κB nuclear factor-κB, PD-L1 programmed cell death ligand 1, PD-L2 programmed cell death ligand 2, PIP2 phosphatidylinositol 4,5-bisphosphate, PIP3 phosphatidylinositol-3,4,5-triphosphate, PI3K phosphatidylinositol 3-kinase, Raf rapidly accelerated fibrosarcoma, Ras rat sarcoma virus, TAK1 transforming growth factor-β-activated kinase 1, TAMs tumor associated macrophages, TNF-α tumor necrosis factor-α, TNFR tumor necrosis factor receptor, Wnt wingless/integrated, Wnt3A Wnt family member 3A, YAP Yes-associated protein, ZEB1 zinc finger E‐box binding homeobox 1, PD-1 programmed cell death protein 1, c-Jun cellular homolog of the viral oncoprotein v-jun, SHP2 Src homology-2 domain-containing protein tyrosine phosphatase-2

A detailed mechanistic outline depicting the proposed signaling pathways involving PD-L1, PD-1, and their interactions with other signaling cascades in cancer is summarized in Fig. 1.

### Critical role of PD-1 in various cancers

#### Breast cancer

In line with its well-known variability in clinical behaviors, breast cancer is a highly heterogeneous disease characterized by distinct molecular subtypes, typically assessed in the routine setting through the expression of HER2, estrogen receptor (ER), and progesterone receptor (PR) [126]. Numerous breast cancer patients carrying germline mutations in the breast cancer 1 (*BRCA1*) or *BRCA2* genes display specific subtypes of the disease [126]. Poly(ADP-ribose) polymerase (PARP) plays an essential role in single-strand DNA repair, and PARP inhibitors have shown anticancer activity against HER2-negative breast cancer cells associated with *BRCA1*/*BRCA2* mutations [126]. Anti-PD-1 therapy or its ligand anti-PD-L1 has been used as monotherapy and has implicated modest response rates in metastatic breast cancer cases [127]. Targeting PD-1 serves as an immune checkpoint to elicit antitumor activity, regulate T cell responses, and assess the presence of PD-L1 in breast cancer cells [128]. The combination of PD-1 inhibition with cyclin-dependent kinase 4/6 inhibitors is significant for treating ER/HER2-positive breast cancer, supported by preclinical data indicating enhanced antitumor activity [126].

#### Cervical cancer

Cervical cancer is a prevalent malignancy among females, ranking as the fourth most commonly diagnosed cancer in women with high mortality rates [129]. However, advancements in screening measures, human papillomavirus (HPV) prophylactic vaccines, removal of precancerous lesions, radical hysterectomy, and chemoradiation, are expected to lead to a decline in both the incidence and mortality of cervical cancer in the future [129]. HPV-mediated immunological response and the potential of ICIs to elicit effective antitumor activity through PD-1-mediated signaling have been reported previously [129]. Elevated expression of PD-1 on Tregs in cervical cancer patients has been found to facilitate the production of TGF-β and IL-10 while inhibiting the production of IFN-γ [130]. In investigations examining the effect of PD-L1 on lymphocytes, blocking PD-L1 resulted in increased proliferation and secretion of IL-10 and TGF-β [130]. Therefore, it can be concluded that inhibiting PD-L1 restoration may improve the T cell-mediated immune responses and enhance antitumor immunity.

#### Colorectal carcinoma (CRC)

CRC is the third most common cancer in the world, with the prognosis determined by tumor stage [131]. The current standard treatment for CRC includes targeted chemotherapy in conjunction with other adjuvant anticancer therapies, such as surgical resection of the tumor [131, 132]. Recently, two key areas of advancement in CRC research have been the immunology-cancer interface and the tumor microenvironment, both of which substantially influence future CRC diagnoses [131]. Studies have reported that in CRC cells, the JAK/STAT signaling pathway triggers the PD-L1 expression, with intrinsic cellular alterations mediating PD-L1 induction in certain contexts [133, 134]. Vascular endothelial growth factor A (VEGF-A) acts as an important ligand involved in the homeostasis maintenance of adult organs and plays a significant role in cancer development [135–137]. Another mechanism that enhances the efficacy of checkpoint inhibition in CRC is the combination of anti-VEGF-A therapy and anti-PD-1 treatment [138]. In CRC cells, immunotherapy can block the PD-1/PD-L1 axis through the gene level downregulation of PD-L1 expression and could be achieved by small interfering RNAs combined with anti-VEGF-A and anti-PD-1 [139].

#### Head and neck cancer

Head and neck cancer is the sixth most prevalent cancer worldwide, arising from various carcinogenic factors such as smoking, alcohol abuse, and HPV infection [140]. Recurrent head and neck squamous cell carcinoma (HNSCC) has a poor prognosis, with an overall survival rate of around 6 to 12 months [141]. Numerous immunotherapeutic methods for the HNSCC are currently under investigation, including tumor vaccines, cell-based therapies, and cytokine therapies. The integration of traditional therapies, such as surgery, radiotherapy, and chemotherapy, with immune checkpoint inhibition, has demonstrated improved effects [140]. Current evidence suggests that almost 45–80% of HNSCC tumors exhibit upregulated PD-L1 levels induced by IFN-γ from NK cells [134, 142, 143]. Studies have also indicated that EGFR/JAK2/STAT1 also functions as a driver for elevated PD-L1 levels in HNSCC [134, 143, 144].

#### Hepatocellular carcinoma (HCC)

HCC is one of the most common types of cancer globally and currently ranks as the third leading cause of cancer-related mortality [145]. Nonetheless, due to the difficulties in detecting symptoms and the physical attributes associated with HCC, curative treatment is not often feasible for over 80% of patients at diagnosis [145]. Overexpression of PD-L1 has also been observed within the HCC microenvironment [146]. In a study including 217 surgically removed HCC samples, elevated levels of PD-L1 were identified in neoplastic cells and intra-tumoral inflammatory cells, correlating with tumor aggressiveness [147]. Another investigation into HCC tissues revealed that PD-L1 expression is significantly associated with high levels of CD8^+^ tumor-infiltrating lymphocytes. Consequently, targeting the PD-1/PD-L1 pathway presents a promising strategy for managing HCC through various ICIs [146]. An additional in vivo study found that the mRNA expressions of *IRF1* and *IRF2* in human HCC tumors were significantly repressed compared to non-tumor cells [148]. The mRNA expressions of *IRF1*, *IRF2*, and *PD-L1* correlated with HCC tumors. IFN-γ induces *PD-L1* mRNA and protein expression by upregulating IRF1 in mouse and human HCC cells [148]. Previous research on human and murine HCC cells indicates that overexpression of IRF2 downregulates IFN-γ-induced activity from the PD-L1 promoter as well as its protein levels [148].

#### Lung cancer

Lung cancer is the leading cause of cancer incidence and mortality worldwide, with an estimated 2.1 million new cases and 1.8 million deaths reported [149]. Immunotherapy utilizing antibodies to inhibit the interaction between PD-1 and PD-L1 has dramatically improved survival rates for some lung cancer patients [150]. The overexpression of PD-1 on CD8^+^ T cells in NSCLC suggests a reduced production of various cytokines and diminished T cell proliferation. Conversely, there is a notable increase in PD-L1^+^ cells compared to adjacent lung parenchyma, and PD-L1 expression on NSCLC cells also correlates with poor prognosis [151].

#### Oral cancer

Oral squamous cell carcinoma is among the most common malignancies [152]. A study using tumor immune estimation resources revealed a relationship between immune checkpoint genes and cytokines, showing a positive correlation between the expression of PD-1, CTLA-4, and hepatitis A virus cellular receptor 2 with levels of IFN-γ and IL-2 [152]. Blocking PD-1 and CTLA-4 as individual immune checkpoints or jointly targeting both inhibitory pathways can partially restore the biological function of T cells in tumors [153].

#### PC

Pancreatic ductal adenocarcinoma (PDAC) is the second leading cause of cancer-related deaths worldwide [154]. Previous study suggests that PD-L1 overexpression in human pancreatic carcinoma tissues is associated with tumor progression, invasiveness, and poor clinical outcomes [155]. Immunotherapies, such as CTLA-4 and PD-1/PD-L1 inhibitors, are promising treatment options for PDAC and provide relief as compared to surgery or chemotherapy [156, 157]. Histone deacetylase 3 (HDAC3), a class I member of the HDAC family, plays critical roles in various functions such as maintaining endothelial integrity, regulating cell proliferation and migration, and modulating macrophage phenotype. HDAC3 inhibitors are being explored as anticancer agents [158, 159]. HDAC3 regulates PD-L1 expression at the transcriptional level through various transcriptional factors, including STAT3, bromodomain containing 4 (BRD4), and p65, while further inhibiting the STAT3 cascade in cancer cells [160]. Prior research has reported that RGFP966, a specific inhibitor of HDAC3 [161], decreases protein and mRNA levels of *PD-L1* in a time- and concentration-dependent manner in PaCa-2 and BxPc-3 PC cells [160]. A recent study further elaborated that inhibition or depletion of never-in-mitosis-A-related kinase 2 reduces PD-L1 expression in PC cells [162]. It has been also reported that there is a negative association between polo-like kinase 1 and PD-L1 expression in mouse and human PDAC. This relationship involves S758-phosphorylated retinoblastoma protein suppressing NF-κB activity along with subsequent inhibition of PD-L1 transcription [163].

#### Penile cancer

Penile cancer is a rare malignancy, accounting for approximately 26,000 cases every year, with a higher incidence rate observed in developing nations [164–167]. Various factors, including phimosis, chronic inflammation of the penis, smoking, lower socioeconomic status, ultraviolet exposure, and HPV infection, are recognized risk factors for penile cancer [165, 167, 168]. Previous studies have demonstrated a strong correlation between PD-L1 expression and penile cancer, with PD-L1 detected in 40–60% of tumor cells [166, 169–171]. A higher prevalence of PD-L1 has been associated with advanced tumor stage and grade of penile carcinoma [171], as well as regional lymph node metastasis and shorter cancer-specific survival rates [166]. This strengthens the link between miRNA expression and penile cancer. Specifically, miR-218 expression is downregulated in penile cancer patients exhibiting higher levels of HPV infection [172]. A small cohort study involving 30 patients demonstrated that EGFR expression is strongly related to an increased risk of recurrence and poorer prognosis among those diagnosed with penile cancer [173]. An elevated presence of CD8^+^ T cells and forkhead box P3 (Foxp3)^+^ Tregs in the stroma of penile cancer suggests inefficient tissue infiltration, signifying an immune escape of tumor cells [165]. Furthermore, a higher incidence of prostaglandin E2 has been found in penile cancer, contributing to the overall immune escape mechanisms within the tumor microenvironment [165].

#### Hematological malignancies

The PD-L1 expressed in leukemia cells binds to the PD-1 receptor on tumor-infiltrating T lymphocytes, establishing the critical PD-1/PD-L1 axis for regulating immune responses. Therefore, the immune system is unable to reject the tumor, leading to immunological tolerance due to inhibited activation of tumor-reactive T cells and their subsequent death. Tumor cells evade immune surveillance through the PD-1/PD-L1 axis, and checkpoint medications targeting this pathway have been approved for the treatment of lymphomas and many solid tumors [174].

The United States Food and Drug Administration (FDA) has authorized monoclonal antibodies developed clinically to block immunological checkpoints, such as CTLA-4, PD-1, and PD-L1. These agents can be utilized in managing a range of solid and hematologic tumors [175]. In myeloma, the development of PD-1/PD-L1 blockade has been temporarily stopped due to safety concerns that need careful examination to safeguard patients and guide future clinical trials. While there has been limited progress in drug development for leukemias, preliminary findings indicate that PD-1 inhibition in combination with epigenetic therapy may show promise in treating myeloid malignancies [175].

#### Soft tissue sarcoma

The literature on soft tissue sarcoma presents conflicting findings on the association between higher PD-1/PD-L1 expression and overall survival outcomes, whether better or worse [176, 177]. These discrepancies may be attributed to various confounding factors, including sampling bias and differences in antibody clones, thresholds, assay constraints, as well as variability among the histological subtypes analyzed [178]. The expression of PD-L1, as determined by immunohistochemical staining, can aid in predicting responses to immune-modulating therapies. Such treatments may prove beneficial in the management of metastatic sarcomas. The PD-L1 expression varies among carcinomas and melanomas. However, its expression seems to be type-specific for sarcomas. Thus, it is postulated that PD-L1-based therapies may exhibit reduced efficacy for cancers other than sarcomas [179].

## Approved checkpoint inhibitors that block PD-1 checkpoint receptors in cancer

Immune checkpoint blockade therapies have garnered significant attention from researchers worldwide. The unprecedented results obtained with these antibody-based immune therapies in enhancing antitumor T lymphocyte activity have led to clinical developments in cancer drug discovery [180]. The FDA has granted further approval for various drugs targeting this immune checkpoint, exhibiting safer clinical outcomes for cancer patients [180]. Several approved ICIs are discussed in the following sections. A detailed analysis of various drugs and synthetic compounds as PD-1/PD-L1 inhibitors is presented in Table 1 [181–187], while several clinical studies related to drugs targeting PD-1/PD-L1 are summarized in Table 2 [188].

**Table 1:** Various drugs/synthetic compounds as PD-1/PD-L1 inhibitors

Name of the approved drug and companies	Cancer type	Anticancer effect	Mechanism of PD-1 action	Reference
Atezolizumab (Roche)	TNBC, NSCLC	Antiproliferation, prevents antibody dependent cellular cytotoxicity	Binds to PD-L1 and prevents its interaction with PD-1 and CD80 receptors	[181]
Avelumab (Merck KGaA and Pfizer)	Merkel cell carcinoma, urothelial carcinoma, and renal cell carcinoma	Antiproliferation	Inhibits PD-L1/PD-1 interaction, removes T cell suppression, inhibits the interaction of PD-L1 and B7.1 receptors	[182]
Cemiplimab (Regeneron Pharmaceuticals)	CSCC	Antiproliferation	Blocks interaction between PD-1/PD-L1 and prevents immune evasion	[183]
Dostarlimab (GlaxoSmithKline)	dMMR recurrent or advanced endometrial cancer	Antiproliferation, restores immune activity	Binds with and inhibits PD-1, blocks the interaction between PD-L1 and PD-L2, activates T cell functions	[184]
Durvalumab (AstraZeneca)	Extensive-stage small cell lung cancer	Antiproliferation, increased T cell activation	Selectively blocks the interaction of PD-L1 with both PD-1 and CD80	[185]
Nivolumab (Bristol Myers Squibb)	NSCLC, mesothelioma	Antiproliferation	Blocks the receptor activation of PD-L1 and PD-L2, resulting in the release of PD-1-mediated inhibition of the immune response	[186]
Pembrolizumab (Merck & Co)	Melanoma, Hodgkin’s lymphoma	Antitumor activity, antiproliferation, inhibit IFN-γ secretion	Prevents PD-L1/PD-1 binding with ligands, preventing T cell immune surveillance	[187]

*CSCC* cutaneous squamous cell carcinoma, *dMMR* deficient DNA mismatch repair, *IFN-γ* interferon-γ, *NSCLC* non-small cell lung cancer, *PD-1* programmed cell death protein 1, *PD-L1* programmed cell death ligand 1, *PD-L2* programmed cell death ligand 2, *TNBC* triple-negative breast cancer

**Table 2:** Clinical trial data of various drugs targeting PD-1/PD-L1

Drug	Phase	No. of patients	Cancer type	Result/Endpoint	Reference
Aminolevulinic acid + nivolumab	II	20	MPM	Results not reported yet	NCT04400539
Camrelizumab + apatinib and eribulin mesylate	II	46	Breast cancer	Results not reported yet	NCT04303741
Durvalumab + platinum −etoposide	III	268	SCLC	Improvement in survival as compared to other drug combination group	[188]
Durvalumab + pralatrexate and romidepsin	I, II	148	PTCL	Results not reported yet	NCT03161223
Eribulin mesylate + gemcitabine	III	465	UC	Results not reported yet	NCT04579224
Eribulin mesylate + sintilimab	II	30	TNBC	Study in progress; results not reported yet	NCT05402722
Eribulin mesylate + pembrolizumab	II	90	Breast cancer	Combination of pembrolizumab + eribulin didn’t show any improvement in efficacy outcome, compared with eribulin alone in this population	NCT03051659
Eribulin mesylate + pembrolizumab	II	57	Liposarcoma, lkeiomyosarcoma, and sarcoma	Results not reported yet	NCT03899805
Eribulin mesylate + pembrolizumab	II	30	Ovarian carcinosarcoma and uterine carcinosarcoma	Results not reported yet	NCT05619913
Icaritin vs. huachashu	III	312	Advanced HBV-related HCC	Results not reported yet	NCT03236636
Icaritin vs. sorafenib	III	200	HCC	Results not reported yet	NCT03236649
Nivolumab + eribulin	I, II	90	Metastatic breast cancer	Results not reported yet	NCT04061863

*HBV* hepatitis B virus, *HCC* hepatocellular carcinoma, *MPM* malignant pleural mesothelioma, *PTCL* peripheral T cell lymphoma, *SCLC* small cell lung cancer, *TNBC* triple-negative breast cancer, *UC* urothelial carcinoma

### Atezolizumab

Atezolizumab is a non-glycosylated humanized monoclonal immunoglobulin G1 (IgG1) antibody that binds to PD-L1, effectively blocking its interactions with PD-1 and enhancing the antitumor response [181]. Promising results obtained from clinical studies led to the FDA’s accelerated approval of atezolizumab in combination with nab-paclitaxel for locally advanced or metastatic TNBC [181]. Furthermore, a randomized, open-label, phase III trial demonstrated the efficacy of atezolizumab compared to platinum-based chemotherapy in patients with NSCLC, showing improved overall survival rates relative to conventional chemotherapeutic approaches [25].

### Avelumab

Avelumab is a humanized IgG1 anti-PD-L1 antibody that specifically targets tumor cells. It inhibits the interaction between PD-L1*/*PD-1 to promote T cell antitumor potential and alleviate the suppression of T cell activity, which can be assessed by evaluating the release of IFN-γ [189, 190]. Importantly, avelumab does not interact with the PD-L2*/*PD-1 pathway, thus maintaining immune homeostasis [191]. Additionally, avelumab disrupts the interaction between PD-L1 with B7.1, another inhibitory receptor expressed on T cells and antigen-presenting cells. By blocking this interaction in the tumor microenvironment, avelumab reactivates T cells and promotes the production of cytokines, thereby enhancing innate immunity against tumor cells [182].

### Cemiplimab

Cemiplimab is a potent and highly effective humanized mAb directed against PD-1. It has received approval for the treatment of metastatic and locally advanced cutaneous squamous cell carcinoma [183, 192]. Cemiplimab can suppress T cell effector function, facilitating immune evasion. Furthermore, it demonstrates superior clinical efficacy and a favorable safety profile, with lower rates of treatment-related death and discontinuation [183]. The safety profile of cemiplimab is comparable to that of other therapies across various indications. Studies have highlighted the adverse effects associated with treatment discontinuation, which may limit its application because of the toxicity profile [183, 193].

### Dostarlimab

Dostarlimab is another humanized IgG4 mAb that acts as an antagonist to PD-1, blocking its interactions with both PD-L1 and PD-L2. It is indicated for various tumor types and has been approved by the USA and the European Union for treating endometrial cancer [184, 194, 195]. Its molecular weight is around 144 kD, and it targets the PD-1 on the surface of T cells, preventing it from interacting with its ligands, PD-L1 or PD-L2, which is expressed in malignant cells. Adverse reactions associated with dostarlimab include anemia, nausea, diarrhea, urinary tract infection, and vomiting. Ongoing clinical studies are exploring additional potential applications for this drug. A recently released clinical trial revealed that individuals with advanced rectal cancer experienced 100% remission without experiencing any serious adverse effects. Patients with other cancers, including ovarian cancer, melanoma, head and neck cancer, and breast cancer, are still being enrolled in this research trial [196]. The published crystal structure of the extracellular domain of PD-1 in association with the dostarlimab Fab fragment indicates that dostarlimab’s heavy chain interacts directly with PD-1 while sterically obstructing binding by the light chain interference. Conformational rearrangements allow dostarlimab to achieve a concave surface on its heavy chain alongside high affinity binding to its ligand [197].

### Durvalumab

Durvalumab is a humanized IgG1κ mAb that specifically targets PD-L1. This drug has received FDA approval for treating small cell lung cancer in combination with atezolizumab [185, 198]. By blocking the interaction between PD-L1 and both PD-1 and CD80, durvalumab enhances antitumor effects and promotes T cell activation [199, 200]. In mouse models, treatment with durvalumab resulted in decreased tumor size, demonstrating its potential therapeutic efficacy [185]. Furthermore, the most commonly reported adverse events include hypo- and hyper-thyroid events and certain hematological events [185].

### Nivolumab

Nivolumab is a fully humanized IgG4 mAb that inhibits PD-1 by effectively binding to PD-L1 and PD-L2. This interaction results in the production of inhibitory signals, reduced T cell proliferation, cytokine production, and cytotoxic activity, thus dampening the immune response [186]. This drug selectively blocks receptor activation of PD-L1 and PD-L2, alleviating PD-1-mediated inhibition of the immune response [186]. Nivolumab is a well-tolerated therapeutic option indicated for CRC and HCC [201, 202]. Additionally, it is used in adjuvant treatment approaches with ipilimumab for NSCLC [203], and with relatlimab for multiple advanced cancers [204]. Nivolumab has demonstrated an improved safety profile along with enhanced tolerability and suitability profile [186].

### Pembrolizumab

Pembrolizumab is a highly selective humanized IgG4κ mAb with a strong affinity for the PD-1 receptor expressed on cytotoxic T lymphocytes [205]. Upon binding to PD-L1 expressed by tumor cells, the drug induces cytotoxic T cell exhaustion, leading to inhibited proliferation [206, 207]. The PD-1/PD-L1 interaction is blocked by pembrolizumab, thus restoring the cytotoxic activity of T lymphocytes [206]. Combination treatment strategies involving other agents, such as neriparib, are being investigated for ovarian cancer, breast cancer, as well as head and neck cancer with promising results [208, 209]. The combination of ipilimumab and pembrolizumab is used for treating advanced melanoma tumors [207]. Adverse events associated with pembrolizumab include fatigue, hypothyroidism, and varying degrees of pneumonitis, which can be managed through cessation of therapy with pembrolizumab [210].

### Retifanlimab

Retifanlimab is a humanized IgG4 mAb that specifically targets human PD-1. By inhibiting the PD-1/PD-L1/2 pathway, this drug enhances T cell activity and boosts the immune response against cancer cells. In 2023, the FDA granted accelerated approval for retifanlimab to treat adult patients with metastatic or recurrent locally advanced Merkel cell carcinoma (MCC). This regulatory decision was supported by data from the phase II POD1UM-201 trial (NCT03599713), an open-label, multi-regional, single-arm study involving 65 participants with metastatic or recurrent locally advanced MCC who had not received prior systemic therapy for advanced disease. In this clinical trial, treatment with retifanlimab achieved a 52% overall response rate and an 18% complete response rate. As a condition of approval, confirmatory trials must demonstrate clinical benefit for these patients. Immune-related adverse reactions, which may be severe or fatal, can occur in patients treated with retifanlimab. Clinically significant immune-related adverse reactions reported include pneumonitis, colitis, hepatitis, endo-dermatologic adverse reactions, crinopathies, hypophysitis, thyroid disorders, type 1 diabetes mellitus, and nephritis associated with renal dysfunction [211].

### Tislelizumab

Tislelizumab is a humanized IgG4 mAb characterized by high affinity and binding specificity for PD-1. This antibody was engineered to minimize binding to FcγR on macrophages, thereby limiting antibody-dependent phagocytosis, which is a potential mechanism of resistance to anti-PD-1 therapies. In 2024, the FDA approved tislelizumab as monotherapy for adults with unresectable or metastatic esophageal squamous cell carcinoma following prior systemic chemotherapy that did not include a PD-1/PD-L1 inhibitor. The approval was based on data from the phase III RATIONALE 302 study (NCT03430843), which showed that tislelizumab significantly improved overall survival compared to chemotherapy. Immune-related adverse reactions such as pneumonitis and hepatitis, including fatal cases, have been reported during treatment with tislelizumab. Other clinically significant immune-related adverse reactions reported contain endocrinopathies, thyroid disorders, adrenal insufficiency, type 1 diabetes mellitus, myositis, nephritis with renal dysfunction, myocarditis, arthritis, polymyalgia rheumatica, pericarditis, and Guillain-Barré syndrome [212].

### Toripalimab

Toripalimab is a recombinant humanized IgG4 mAb targeted against the PD-1 receptor. It received initial approval in China in December 2018 for the treatment of unresectable or metastatic melanoma in previously treated patients [213]. In October 2023, toripalimab was granted FDA approval for select patients with recurrent or metastatic non-keratinizing nasopharyngeal carcinoma who experienced disease progression on or after platinum-containing chemotherapy; based on positive results from the phase II clinical study POLARIS-02 (NCT02915432) and the phase III clinical trial JUPITER-02 (NCT03581786) [214]. Toripalimab belongs to a next-generation PD-1 checkpoint inhibitor with potential applicability for treating cancer patients regardless of their PD-L1 status. This antibody binds to the FG loop of PD-1 and promotes Th1- and myeloid-derived inflammatory cytokine responses in healthy human peripheral blood mononuclear cells (PBMCs) in vitro. In a TCR activation-dependent system, SEB-stimulated PBMCs demonstrated increased levels of IFN-γ, IL-2, TNF, granulocyte–macrophage colony-stimulating factor, and IL-18, indicating a strong Th1 response. Additionally, the binding of toripalimab to PD-1 induced reduced recruitment of SHP1 and SHP2, the negative regulators of T cell activation, in Jurkat T cells ectopically expressing PD-1 [215]. Immune-mediated adverse events with toripalimab consist of pneumonitis, colitis, hepatitis, endocrinopathies, nephritis accompanied by renal dysfunction, and skin adverse reactions.

### Limitations with available drugs

Despite the availability of various therapeutic agents, significant concerns remain regarding their associated adverse events. For instance, organ-specific and multi-organ immune-related adverse events (irAEs) are common among cancer patients. In trials involving NSCLC, a post hoc pooled analysis revealed that 5.4% of patients treated with atezolizumab experienced multiorgan irAEs. Furthermore, it was reported that out of 1548 patients, 424 individuals (27%) experienced a lot of 730 recorded irAE events [216]. In a case study, a 75-year-old man with stage IV urothelial carcinoma developed ICI-induced diabetes mellitus [217], leading to the suspension of treatment. A recent comprehensive pharmacovigilance analysis indicated that atezolizumab, pembrolizumab, nivolumab, avelumab, cemiplimab, durvalumab, ipilimumab, as well as combinations such as nivolumab/ipilimumab and pembrolizumab/ipilimumab all exhibited substantial reporting signals for immune thrombocytopenia. Patients receiving concurrent anti-PD-1 and cytotoxic T cell-associated protein-4 therapy are at an increased risk for ICI-induced immune thrombocytopenia [218].

Thus, while ICIs demonstrate significant potential and efficacy in cancer treatment, their associated adverse events cannot be overlooked. Therefore, natural compounds may be utilized either alone or in combination as adjuvants to increase the effectiveness of these therapies or to mitigate their adverse effects. The following section provides a detailed overview of a broad range of natural compounds that can target PD-1/PD-L1 in cancer therapeutics.

## Natural compounds targeting PD-1/PD-L1 in cancer

Natural products exhibit a plethora of beneficial properties, some of which are extensively employed in the treatment of various diseases. Secondary metabolites derived from plant sources are examples that have shown antitumor activity. This section provides a critical evaluation of diverse natural compounds regarding their effects on PD-1/PD-L1 in both preclinical and clinical studies related to cancer. A comprehensive literature search was conducted using several databases, including PubMed, Scopus, ScienceDirect, and Google Scholar. Various keywords were utilized, such as “natural compounds” AND “cancer” AND “PD-1” or “PD-L1”, with more specific terms like “biological products” OR “biological” AND “products” OR “biological compounds” OR “natural” AND “compounds” OR “natural products” AND “cancers” OR “cancerated” OR “canceration” OR “cancerization” OR “cancerized” OR “cancerous” OR “neoplasms” OR “neoplasms” OR “cancer” OR “cancers” AND “PD-1” OR “PD-L1” OR “programmed cell death 1 receptor” OR “programmed cell death protein 1”. Additionally, individual searches for natural compounds were performed using the “AND” operator along with “PD-1” OR “PD-L1” AND “cancer”. There was no time limit imposed on published studies, and the final search was conducted in May 2023. The clinical data were retrieved from https://clinicaltrials.gov/ in August 2024. A detailed discussion based on in vitro and in vivo studies examining the influence of various natural compounds on PD-1/PD-L1 in cancer is presented in Tables 3 [219–248] and 4 [220, 222, 224, 226, 227, 230–232, 235–237, 240, 241, 243, 244, 246, 247, 249–254], respectively.

**Table 3:** In vitro anticancer effects of bioactive natural compounds mediated through modulation of PD-1/PD-L1 pathway

Natural compounds	Concentration	Cell line used (cancer type)	Anticancer effect	PD-1/PD-L1 related mechanisms	Reference
Apigenin	30 μmol/L	MDA-MB-468 SK-BR-3 (breast cancer)	↓Proliferation	↓STAT1 activation, ↓IFN-γ and IFN-β induced PD-L1 expression, ↓T cell activity, ↓IL-2 synthesis	[219]
Berberine	5–20 μmol/L	H460, H1975, H358, HCC827 A549, H1299 (lung cancer)	$\uparrow$ T cell immunity	↓IFN-γ-induced PD-L1 expression, ↓PD-1 binding to cancer cells, ↑caspase-3, ↑caspase-7, ↑activation of T cells, protein degradation through Ub/proteasome pathway	[220]
Britannin	30 μmol/L	A549, HeLa, Hep3B, and HUVEC, HCT116 (CLC)	↓Proliferation, ↓angiogenesis	↓PD-L1 activity, ↓HIF-1α nuclear protein expression, ↓mTOR/p70S6K/4EBP1, ↓interaction between HIF-1α and c-Myc, ↓EPO, ↓cyclin-D1, ↓VEGF, ↓MMP-9	[231]
Cinnamaldehyde	50–100 μmol/L	MUTZ-3 (leukemia)	Not reported	↑PD-L1 expression, ↑CD86 expression	[242]
Cordycepin	0.01–10 μmol/L	4NAOC-1 (OC)	↓Colony formation, ↓cell proliferation, ↓cell cycle of G2/M phase	↓PD-L1, p-EGFR, p-ERK1/2, p-STAT3, p-p70 S6K, β-catenin, cyclin B1 expression	[243]
Curcumol	0.1–30 μmol/L	Hep3B, A549, HeLa (HC)	↓Proliferation, ↓invasion	↓*PD-L1* mRNA and protein expression, ↓STAT3 phosphorylation by suppressing JAK1, JAK2 and Src kinase expression, ↓HIF-1α accumulation and expression, ↓mTOR/p70S6K/eIF4E and MAPK signaling pathways	[244]
Epigallocatechin gallate	10 μmol/L	1205Lu, A375 HS294T, melanoma	↑Antitumor effects by reactivation of T cells	↓PD-L1 and PD-L2 levels ↓IFN-γ-mediated *STAT1* mRNA expression, ↓*IRF1* mRNA expression, ↓JAK/STAT signaling	[245]
Erianin	1–100 μmol/L	HeLa, HCT-116, Hep3B A549 (CC)	↓Proliferation, ↓angiogenesis, ↑T cell activity	↓*PD-L1* mRNA levels Induces functional lysosome biogenesis, ↓HIF-1α, ↓mTOR/p70S6K/eIF4E ↓Ras protein expression, ↓cyclin D1, ↓ICAM-1, induces G1 phase arrest Restores T cell activity, ↓Interaction between PD-1 and PD-L1, ↓VEGF, ↓MMP-9	[246]
Evodiamine	2–20 μmol/L	H1975, H1650, H2228, HCC827 (NSCLC)	↑Apoptosis, ↓proliferation and cell cycle arrest at the G2 phase	↓MUC1-C, ↓IFN-γ-induced PD-L1 expression, ↑CD8^+^ T cells	[247]
Fraxinellone	100 μmol/L	A549, HeLa, HCT116, Hep3B, HLF-a (HC)	↓Proliferation, ↓angiogenesis	↓PD-L1 expression, ↓STAT3 phosphorylation and nuclear translocation, ↓JAK1, JAK2, Src phosphorylation ↓accumulation of HIF-1α, ↓mTOR/p70S6K/eIF4E, MAPK signaling	[248]
Gallic acid	100–400 μmol/L	A549, H292 (lung cancer)	↓Proliferation, ↑apoptosis	↓*PD-L1* mRNA protein expression, ↓PI3K/Akt phosphorylation, ↑p53 dependent miR-34a expression	[221]
Ginsenoside Rk1	100–125 μmol/L	A549, PC9 (lung cancer)	↑Cell death, ↑apoptosis	↓PD-L1 expression, interference with NF-κB activity, ↑G1 phase arrest	[222]
Hesperidin	20–200 μmol/L	MDA-MB-231 (breast cancer)	↓Proliferation, ↑apoptosis	↓*PD-L1* mRNA and protein expression, ↓Akt, ↓NF-κB signaling, ↓MMP-9, ↓MMP-2	[223]
Icaritin	0.25–1 μmol/L	B16F10, HL-60 (melanoma)	↓T cell activity	↓PD-L1 expression, ↓TNF-α, ↓IL-6	[224]
Kaempferol	5–20 μmol/L	Jurkat cells, CHO-K1 (OVC)	↓Proliferation, ↑apoptosis	↓PD-1/PD-L1 interaction	[225]
Licochalcone A	10–50 μmol/L	HCT116, HeLa, A549, Hep3B HCT116 (CLC)	↓Proliferation, ↑apoptosis	↓PD-L1 expression, ↓TNF-α-induced p65, IKKα/β phosphorylation, TRAF2 and R1P1 expressions and nuclear translocation, ↓Ras/Ref/MEK, ↓interaction between p65and Ras Restores T cell antitumor activity, ↓PD-1 and PD-L1 interaction, ↓c-Myc, ↓cyclin-D1, ↑cleaved PARP, ↑cleaved caspase-8	[226]
Luteolin	10–50 μmol/L	H358, H460, A549 (lung cancer)	↓Cell growth, ↑apoptosis	↓PD-1 and PD-L1 interactions, ↓p-STAT3, ↓IL-2	[227]
Lycopene	4–25 μmol/L	CAL27, SCC9, SCC25 (tongue carcinoma)	↓Proliferation, ↑apoptosis	↓PD-L1 signaling, ↓IGF-1R, p-Akt1, ↓NF-κB/STAT3/c-Myc	[228]
Myricetin	1.25–80 µmol/L	A549, MDA-M-231, NCI-460 (lung cancer and Breast cancer)	↓Proliferation, ↑apoptosis	↓IFN-γ-mediated PD-L1 expression, ↓IDO1, ↓JAK/STAT, ↓IRF1 activation	[229]
Panaxadiol	1–30 μmol/L	HCT116, SW620, HT29 (CLC)	↓Proliferation	↓PD-L1 protein expression, ↓HIF-1α nuclear accumulation, ↓STAT3 expression, ↓p-JAK1, ↓p-JAK2, ↓Src expression	[230]
Polydatin	50–150 μmol/L	Caco-2, HCT116 (CLC)	↓Proliferation, ↑apoptosis	↓PD-L1 expression, ↓miR-382, ↓cleaved caspase-3, ↓cleaved caspase-9 protein	[232]
Quercetin	5 μmol/L	HEK293, MDA-MB-231, NCI-460 (breast cancer and lung cancer)	↑T cell activity	↓PD-1/PD-L1 binding	[233]
Resveratrol	50–100 μmol/L	Cal51, HCT116 (breast cancer and CRC)	Cell cycle arrest in the S phase, ↑apoptosis	↓PD-L1 expression, ↓NF‑κB	[234]
Romidepsin	10 μmol/L for CT26 cells, 40 nmol/L for MC38 cells	CT26, MC38 (CLC)	↓Proliferation, ↑apoptosis, ↓cells in S + G2/M phase	↑PD-L1, ↑acetylation of histone H3/H4, ↑cleaved caspase-3	[235]
Sativan	1–100 μmol/L	MDA-MB-231, BT549 (breast cancer)	↓Proliferation, ↑apoptosis	↓PD-L1, ↑miR-200c, ↓EMT	[236]
Shikonin	1–5 µmol/L	PANC-1, BxPC3 (PC)	↓Immune evasion, ↑killing of PC cells	↑PD-L1 degradation, ↓NF-κB/STAT3/CSN5, ↓cleaved-caspase3	[237]
Silibinin	50–100 μmol/L	A549, H292, H460 (lung cancer)	↓Proliferation, ↑apoptosis	↓PD-L1 protein expression, ↓JAK2/STAT5 phosphorylation, ↓PI3K/Akt, ↓p-Akt, ↓MMP-2	[238]
Silibinin	25–200 µmol/L	C666-1, (nasopharyngeal carcinoma)	↓Apoptosis	↓PD-L1 expression, ↓HIF-1α, ↓lactate synthesis	[239]
Triptolide	5–80 nmol/L	JCRB0260 (ORC)	↓Proliferation	↓PD-L1 expression, ↓IFN-γ-mediated PD-L1 expression, ↓JAK2	[240]
β-elemene	10–20 μg/ml	TE-1, KYSE-150 (esophageal cancer)	↓Proliferation, ↑apoptosis	↓PD-L1 expression, ↓ p-Akt, ↓cleaved caspase-3, ↓cleaved PARP	[241]

*↓* inhibits/inhibited/inhibit/inhibiting/downregulation/downregulating/decrease/decreased/downregulates, *↑* increased/enhanced/upregulated/upregulation. *CC* cervical cancer, *CLC* colon cancer, c-Myc cellular myelocytomatosis oncogene, *CRC* colorectal cancer, *CSN5* constitutive photomorphogenic-9 signalosome 5, *ERK* extracellular signal-related kinases, *EMT* epithelial-to-mesenchymal transition*, eIF4E* eukaryotic translation initiation factor 4E*, EPO* erythropoietin, *4EBP1* eukaryotic initiation factor 4E-binding protein 1, *HC* hepatic cancer, *HIF-1α* hypoxia inducible factor-1α, *HUVEC* human umbilical vein endothelial cell, *ICAM-1* intercellular cell adhesion molecule-1, *IDO1* indoleamine 2,3-dioxygenase 1, *IFN-γ* interferon-γ, *IFN-β* interferon-β, *IGF-1R* type 1 insulin-like growth factor receptor, *IL*-*2* interleukin-2, *IL*-*6* interleukin-6, *IRF1* interferon regulatory factor 1, *JA*K Janus kinase, *MAPK* mitogen-activated protein kinase, *MEK* mitogen-activated extracellular signal-regulated kinase, *MMP* matrix metalloproteinase, *mTOR* mammalian target of rapamycin, MUC1-C, *NF-κB* nuclear factor-κB, *NSCLC* non-small cell lung cancer, *OC* ovarian cancer*, ORC* oral cancer, *OVC* ovarian cancer, *PARP* poly(ADP ribose) polymerase, *PC* pancreatic cancer, *PD-1* programmed cell death protein 1, *PD-L1* programmed cell death ligand 1, *PD-L2* programmed cell death ligand 2, *PI3K* phosphatidylinositol 3-kinase, *Ras* rat sarcoma virus, *Ref* rapidly accelerated fibrosarcoma, *RIP1* receptor interacting protein kinase 1, *STAT* signal transducers and activators of transcription, *TNF-α* tumor necrosis factor-α, *TRAF2* TNF receptor associated factor-2, *VEGF* vascular endothelial growth factor

**Table 4:** In vivo antitumor effects of bioactive natural compounds mediated through modulation of PD-1/PD-L1 pathway

Natural compounds	Dose	Animal model used (cancer type)	Antitumor effects	PD-1/PD-L1 related mechanisms	Reference
(-)-Sativan	25 or 50 mg/(kg·d)	MDA-MB-231 mouse xenograft (breast cancer)	↓Tumor development	↑miR-200c, regulates miR-200c/PD-L1 expression	[236]
5-aminolevulinic acid	100 mg/kg	B16F10 mouse model (melanoma)	↓Tumor growth, ↑intratumoral efficacy of T cells	↑PD-L1 blockade	[249]
Apigenin	30 mg/kg	Human KRAS-mutant & Lewis tumor-bearing mice (lung cancer)	↓Tumor growth, ↓apoptosis	↓STAT3 phosphorylation, ↓IFN-γ-, PD-L1	[227]
Berberine	4 or 8 mg/kg	Lewis’s tumor-bearing mice (lung cancer)	↑Antitumor immunity	↑Cleaved caspase 3, ↓PD-L1, ↓Ki-67	[220]
Britannin	5 or 15 mg/kg	Murine xenograft model (CLC)	↓Tumor growth, ↓tumor size	↓HIF-1α expression, ↓PD-L1 protein synthesis	[231]
Cordycepin	200 or 500 mg/kg	Murine OC model	↓Tumor growth, ↓proliferation	↓PD-L1, p-EGFR, p-ERK1/2, Ki-67, p-STAT3, p-p70S6K, β-catenin, cyclin B1 expression, ↑p-STAT1 expression, ↓IL-17A levels	[243]
Curcumin	5 mg/kg	Lewis tumor-bearing mice (human OC)	↑Antitumor T cell responses	↓CSN5, ↓PD-L1 expression through STAT3	[250]
Curcumol	30 mg/kg	Hep3B xenograft model (HC)	↓Tumor growth	↓HIF-1α, PD-L1, VEGF, pSTAT3	[244]
	50 mg/kg	Lewis tumor-bearing mice (HC)	↓Proliferation, ↓invasion, ↓metastasis	↓p-STAT3, ↓HIF-1α protein synthesis	[244]
Erianin	25 or 75 mg/kg	HeLa mouse xenograft (CC)	↓Tumor growth, ↓angiogenesis, ↓proliferation	↓Ras, PD-L1, VEGF and HIF-1α expression, ↓PD-L1 expression	[246]
Evodiamine	10, 20, or 30 mg/kg	Female H1975 xenograft nude mice (NSCLC)	↓Tumor growth, ↑apoptosis, ↓proliferation	↓MUC1-C, ↓IFN-γ-induced PD-L1 expression, ↑CD8^+^ T cells	[247]
Fucoidan 4	200 or 400 mg/kg	Breast cancer-bearing rats	↓Tumor incidence, ↓tumor weight	↓Foxp3, PD-L1 expression, ↓p-PI3K, p-Akt ↓CD3^+^Foxp3^+^ Tregs	[251]
Ginsenoside Rk1	20 mg/kg	BALB/c athymic nude mice (lung cancer)	↓Tumor growth, ↓tumor volume	↓PD-L1 expression by targeting the NF-κB pathway	[222]
Icaritin	70 mg/kg; 35 mg/kg	B16F10 tumor-bearing mice (CRC)	↓Tumor growth	↓PD-L1 expression ↓frequency of CD11b^+^Gr1^+^ MDSCs and PD-L1 expression	[224]
Licochalcone A	15 or 50 mg/kg	HCT116 xenograft model (CLC)	↓Proliferation, ↑apoptosis	↓*PD-L1* protein and mRNA expression, ↓TNF-α-induced p65, IKKα/β phosphorylation, TRAF2, RIP1, c-Myc, and cyclin D1 expression, ↓NF-κB, Ras/Raf/MEK signaling, ↓TNF-α-induced p-MEK and p-Raf expressions	[226]
Luteolin	30 mg/kg	Human KRAS-mutant & Lewis tumor-bearing mice (lung cancer)	↓Tumor growth, ↓apoptosis	↓STAT3 phosphorylation, ↓IFN-γ, PD-L1	[227]
Lycopene	40 mg/kg	Nude mouse (lung cancer)	↓Tumor volume, ↓tumor weight	↑IL-1, IFN-γ levels, ↓IL-4 and IL-10 levels, ↑CD4^+^/CD8^+^ ratio, ↑CD8^+^ T cells, ↓PD-L1 expression	[252]
Mithramycin A	1.5 mg/kg	C57BL/6 mice orthotopic murine model (CRC)	↓Tumor growth	↓g-MDSCs, M2 macrophages	[253]
Panaxadiol	10 or 30 mg/kg	BALB/c athymic nude mice (CLC)	↓Tumor growth, ↓proliferation	↑Lysis of HCT116 cells by T cells, ↓PD-L1 expression	[230]
Polydatin	150 mg/kg	Orthotropic xenograft mouse model (CRC)	↓Tumor growth, ↑apoptosis	↑Expression of miR-382, ↓PD-L1 expression	[232]
Romidepsin	1 mg/kg	C57BL/6 mice model injected with CT26 cells (CLC)	↓Tumor growth, ↑apoptosis	↑PD-L1, ↑acetylation of histone H3/H4	[235]
Shikonin	5 mmol/L	Tumor-bearing model in mice (PC)	↓Tumor weight, ↓tumor volume	↓PD-L1 glycosylation, ↓NF-κB/STAT3 and NF-κB/CSN5	[237]
Trabectedin	0.1 mg/kg	ID8 ovarian tumor mice (OVC)	↓Tumor growth	↑IFN-γ-induced PD-L1 expression	[254]
Triptolide	0.15 mg/kg	PDTX OC tissues, PDTX-mice	↓Tumor growth	↓JAK2 protein synthesis, ↓PD-L1	[240]
β-elemene	100 mg/kg	Nude mice (EC)	↓Tumor volume, ↓tumor weight, ↑apoptosis, ↓tumor migration, ↓proliferation	↓Expression of p-Akt and PD-L1	[241]

↓ inhibits/inhibited/inhibit/inhibition/downregulated/downregulation/downregulating/decrease/decreased; ↑ increased/enhanced/upregulated/upregulation. *CC* cervical cancer, *CD* cluster of differentiation, *CLC* colon cancer, *CRC* colorectal cancer, *CSN5* constitutive photomorphogenic-9 signalosome 5, *EGFR* epidermal growth factor receptor, *ERK* extracellular signal-related kinase, *Foxp3* forkhead box protein 3, *HC* hepatic cancer, *HIF-1α* hypoxia inducible factor-1α, *IFN-γ* interferon-γ, *IKKα* inhibitor of nuclear factor-κB kinase α, *IKKβ* inhibitor of nuclear factor-κB kinase β, *IL* interleukin, *JAK* Janus kinase, *MDSCs* myeloid derived suppressor cells, *MEK* mitogen-activated extracellular signal-regulated kinase, *MUC1-C* mucin 1 C-terminal subunit, *NF-κB* nuclear factor-κB, *OC* oral cancer, *OVC* ovarian cancer, *PC* pancreatic cancer, *PDTX* patient derived tumor xenograft, *PD-1* programmed cell death protein 1, *PD-L1* programmed cell death ligand 1, *PI3K* phosphatidylinositol 3-kinase, *Ras* rat sarcoma virus, *Ref* rapidly accelerated fibrosarcoma, *RIP1* receptor interacting protein kinase 1, *NSCLC* non-small cell lung cancer, *STAT* signal transducers and activators of transcription, *TNF-α* tumor necrosis factor-α, *TRAF2* TNF receptor associated factor-2, Tregs regulatory T cells, *VEGF* vascular endothelial growth factor

### Plant secondary metabolites (pure phytochemicals)

#### Alkaloids

**Fig. 2:**
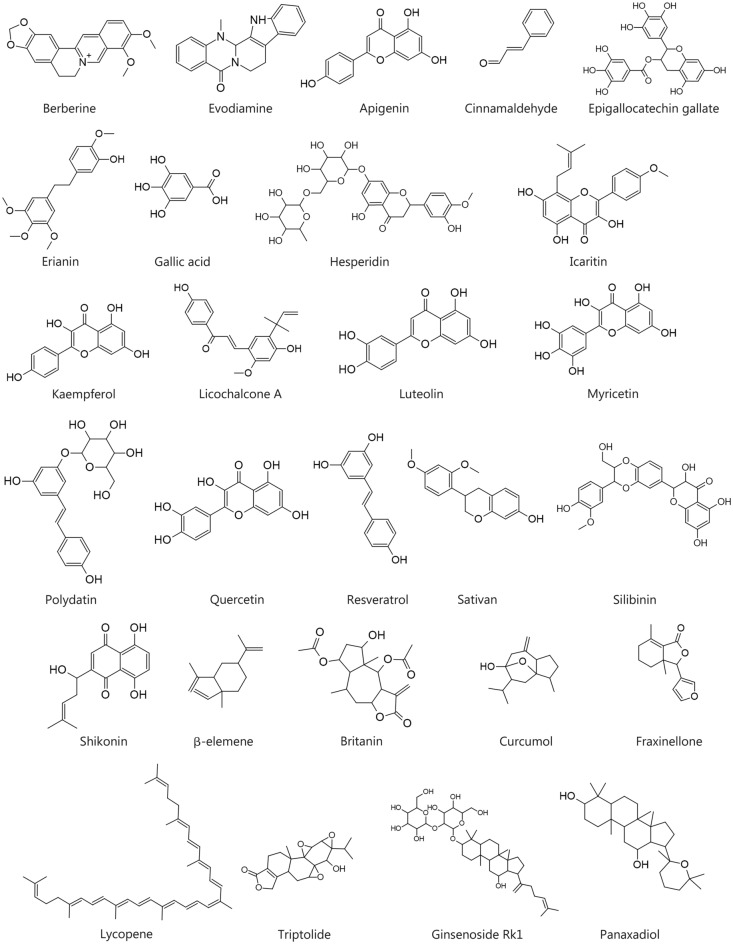
Chemical structures of bioactive anticancer phytochemicals targeting PD-1/PD-L1

**Berberine.** Berbarine is obtained from the rhizomes of *Coptis chinensis* Franch. and *Hydrastis canadensis* L. and also found in various plant parts, such as *Berberis*, *Mahonia, Hydrastis,* and *Coptis genera* [255] (Fig. 2)*.* This compound belongs to the category of quaternary isoquinoline alkaloidal, and has demonstrated promising efficacy in treating a range of conditions, including carcinomas, bacterial infections, diabetes, high cholesterol, cardiovascular diseases, inflammatory disorders, and diarrhea [256]. Research indicates that berberine influences tumor cell cycle, induces cell apoptosis, and inhibits cellular proliferation, thus presenting potential avenues for future antitumor therapies [256]. In a study assessing the effectiveness of berberine across different cell lines, it was observed that berberine induces a concentration-dependent decrease in PD-L1 expression while enhancing T cell cytotoxic in diverse cancer cell lines such as H460, H1975, H358, H157, and HCC827 [220].

In a tumor microenvironment, MDSCs and Tregs primarily mediate the inhibition of T cell activity [257, 258]. Cytometric analysis revealed that MDSCs and Tregs exhibit a reduced frequency in berberine-treated C57BL/6 mice bearing subcutaneous Lewis tumor. The antitumor effects of berberine were also demonstrated, suggesting an increase in intratumoral T cell infiltration alongside enhanced levels of antitumor T cells [220]. Loss of antitumor efficacy observed in nude mice with T cell deficiency further indicates downregulation of PD-L1 and Ki-67 at the posttranslational level accompanied by increased levels of cleaved caspase-3 [220]. Additionally, regulation of PD-L1 by constitutive photomorphogenic-9 signalosome 5 (CSN5) has been established [259]. This regulatory mechanism is elucidated through a molecular docking model involving berberine with CSN5. The binding observed at the Glu76 residue during molecular docking suggests a potential interaction that may inhibit CSN5, thereby regulating PD-L1 levels [220]. Further evidence also reveals that ubiquitination of PD-L1 mediated by berberine results in protein degradation, confirming that PD-L1 degradation occurs via the proteasome pathway [220].

From an immunological perspective, berberine functions as an antitumor agent. It selectively binds to glutamic acid 76 of CSN5, thereby inhibiting the PD-1/PD-L1 axis through deubiquitilation and degradation of PD-L1 [220]. Berberine and its derivatives are also regarded as promising therapeutic agents for managing NSCLC. Furthermore, berberine can be administered in combination with other medications such as osimertinib [260]. These findings indicate that berberine inhibits tumor growth via multiple distinct pathways [261].

**Evodiamine**. Evodiamine, a quinozole alkaloid derived from the fruit of *Tetradium ruticarpum* (A. Juss.) T.G. Hartley [synonym *Evodia ruticarpa* (A. Juss.) Hook. f. & Thomson], has been traditionally used in Chinese medicine for the treatment of cancer, metabolic disorders, autoimmune conditions, and inflammatory diseases [262] (Fig. 2). Evodiamine showed concentration-dependent inhibition of cell growth and apoptosis with a decrease in expression of *PD-L1* mRNA and protein, as well as mucin 1 C-terminal subunit (*MUC1-C*) mRNA in H1650 and H1975 cell lines. Evidence suggests that it inhibits IFN-γ-induced PD-L1 protein expression [247]. Western blotting analysis revealed the downregulation of MUC1-C after evodiamine treatment, with similar trends observed in the LLC model, where tumor growth and volume were suppressed. Post-treatment with evodiamine resulted in an increased amount of CD8^+^ T cells along with enhanced activity [247]. Additionally, evodiamine has been shown to exert antitumor effects against esophageal squamous cell carcinoma through cell cycle arrest during the M phase via the p53/p21 signaling pathway [263].

Moreover, evodiamine reduced the expression of glutathione peroxidase 4 (GPX4), leading to ferroptosis. This process can be inhibited by the ferroptosis inhibitor ferrostatin-1. Additionally, evodiamine impeded angiogenesis by upregulating the expression of Semaphorin-3A (Sema3A). In prostate cancer cells, lactate treatment boosted the expressions of hypoxia-inducible factor-1α (HIF-1α) and PD-L1 while concurrently reducing Sema3A expression. This effect could be mitigated by inhibiting the expression of monocarboxylate transporter 4. Furthermore, evodiamine significantly suppressed lactate-induced angiogenesis in prostate cancer cells by limiting histone lactylation and HIF-1α expression, which improved Sema3A transcription while downregulating PD-L1 transcription.

Evodiamine significantly reduced the growth of prostate cancer xenografts in nude mice in vivo by suppressing the expressions of HIF-1α, lactylation of histone 3 on lysine residue 18 (H3K18la), GPX4, PD-L1, and proliferation markers. Additionally, it increased the expression of Sema3A, which effectively prevented angiogenesis. Consequently, evodiamine may function as a metabolic-epigenetic regulator and represents a promising candidate for antiangiogenic therapy or immunotherapy in prostate cancer. Furthermore, Sema3A is identified as a crucial antineoplastic biomarker [264]. In B16-F10 cells and tumors, evodiamine inhibited PD-L1 and proteins associated with the PI3K/Akt signaling pathway. These findings imply that evodiamine might be an effective therapeutic agent for treating melanoma and may confer additional antitumor benefits by enhancing the immune environment of the cancer [265].

#### Phenolic compounds

**Apigenin**. Apigenin, a natural flavone (4’,5,7-trihydroxyflavone), is found in varying proportions in onions, grapefruits, oranges, parsley, and various common fruits and vegetables [266, 267] (Fig. 2). At elevated concentrations, it has been shown to induce apoptosis, G2/M cell cycle arrest, and inhibit the proliferation of breast cancer cells [219, 268]. Apigenin inhibited IFN-γ and IFN-β induced expression of PD-L1 at a concentration of 30 μmol/L in breast cancer cells and human mammary epithelial cells [219, 269, 270]. In melanoma cell lines A375, A2058, and RPMI-7951, apigenin resulted in decreased G1 and S phase cell counts alongside decreased levels of IFN-γ and STAT1 phosphorylation while enhancing T cell activity and upregulating IL-2 levels [219, 271]. A study investigating apigenin as an anticarcinogenic agent in KRAS-mutant lung cancer cells revealed its ability to inhibit MUC1-C and PD-L1 expression, accompanied by the downregulation of STAT3 expression [227].

In vivo experiments utilizing H358 tumor xenografts, LLC, and KRAS mutant NSCLC models indicated apigenin treatment effectively inhibited tumor growth. These findings correlated with results from a combination therapy involving apigenin and PD-1 mAb, which showed reductions in tumor volume, weight, size, and lung lesions [227]. Apigenin decreased the expression of PD-L1 in dendritic cells, increasing the cytotoxicity of cocultured cytokine-induced killer cells against melanoma cells. Furthermore, apigenin inhibited the growth of melanoma, and its reduction of PD-L1 expression exerted a dual effect on both antigen-presenting cells and tumor cells [271].

**Cinnamaldehyde**. Cinnamaldehyde, the active constituent of the bark from the *Cinnamomum cassia* Nees ex Blume tree [272], is an aromatic aldehyde widely distributed in various analogs [273] (Fig. 2). It exhibits a range of properties, such as antibacterial [272], antifungal [274], antidiabetic [275], and immunomodulatory effects. Notably, cinnamaldehyde possesses antiproliferative potential against multiple tumor types and has been utilized to induce apoptosis in cancerous cells [276]. Human acute myeloid leukemia MUTZ-3 cells were utilized to assess the potential effect of cinnamaldehyde on PD-L1 expression and observed that at varying concentrations, cinnamaldehyde led to upregulation of PD-L1 expression [242]. Additionally, treatment with cinnamaldehyde resulted in a reduction in monocyte chemoattractant protein 1 (MCP-1), a soluble factor released from MUTZ-3 cells [242]. The presence of CD86 expression in PD-L1 has been previously identified, and it was observed that there was an increase in CD86-expressing MUTZ-3 cells upon treatment with different concentrations of cinnamaldehyde. This observation serves as substantial evidence supporting the notion that cinnamaldehyde upregulates PD-L1 expression [242].

**Epigallocatechin gallate (EGCG)**. EGCG, the most prevalent catechin found in tea, is derived from the leaves and buds of *Camellia sinensis* (L.) Kuntze (Fig. 2). It possesses numerous health benefits such as antitumor, antiobesity, antidiabetic, as well as neuroprotective effects [277–279]. In vitro experiments have indicated that EGCG treatment led to the transcriptional inhibition of IFN-γ mediated upregulation of PD-L1 and PD-L2 across all tested melanoma cell lines including 1205Lu, A375, and HS294T [245]. EGCG also showed downregulation of STAT1 expression and its target *IRF1* mRNA levels, indicating that the downregulation of PD-L1 and PD-L2 by EGCG was mediated through the inhibition of the JAK/STAT pathway [245]. Furthermore, EGCG exhibited inhibitory effects on glycogen synthase kinase**-**3β (GSK-3β) phosphorylation while promoting GSK-3β expression and reducing β-catenin levels in CRC and breast cancer cell lines [280]. Moreover, EGCG promoted NF-κB-mediated upregulation of miR-155, directly targeting MDR1 in CRC cell lines [280]. Similar findings were observed in lung cancer cell lines where the expression of PD-L1 mediated by IFN-γ was downregulated following EGCG treatment. This effect was primarily attributed to the inhibition of the JAK2/STAT1 signaling pathway [280]. Research revealed that EGCG inhibited the JAK/STAT signaling pathway in melanoma, thereby leading to decreased expression of PD-L1. Consequently, these cytotoxic T lymphocytes were reactivated to sustain an antitumor immune response due to the elimination of PD-L1-mediated immunoinhibitory signals between tumor cells and cytotoxic T lymphocytes (CTLs) in the tumor microenvironment [281].

In vitro experiments have demonstrated that EGCG administration inhibited JAK/STAT signaling induced by IFN-γ and the generation of immune checkpoint molecules, specifically PD-L1 and PD-L2. This effect was observed in both human and animal melanoma cells, resulting from a reduction in STAT1 expression and phosphorylation, which subsequently decreased the activity of the regulator IRF1 for PD-L1/PD-L2. It was demonstrated that CD8^+^ T cells mediated the in vivo tumor-inhibitory action of EGCG, and the result was similar to that of anti-PD-1 therapy in mice. However, their mechanisms of action differed somewhat. T cell reactivation was caused by EGCG’s ability to reduce PD-L1 expression and inhibit JAK/STAT signaling in tumor cells, whereas anti-PD-1 therapy interrupted the interplay between PD-1 and PD-L1. Finally, research has shown that EGCG suppresses JAK/STAT signaling in melanoma, enhancing antitumor immune responses [245, 282].

**Erianin**. Erianin, a natural compound obtained from *Dendrobium Chrysotoxum* Lindl*.,* has been used as a traditional Chinese herbal medicine and is reported to exert anti-inflammatory, anticataract, antioxidant, and antihyperglycemic effects, along with antitumor potential against various cancerous cells [283, 284] (Fig. 2). In vitro study on cervical cancer cell lines showed concentration-dependent inhibition of *PD-L1* protein expression and corresponding mRNA levels by erianin [246]. The autophagy lysosomal pathway serves as an important degradation system for removing unused proteins and exhibits varied interactions with erianin. It was observed that erianin promoted the transport of PD-L1 to lysosomes for subsequent degradation, indicating that erianin enhanced lysosomal activity further promoting the degradation and proteolysis of PD-L1 [246]. Previous findings suggested some common links between the inhibition of PD-L1 expression and the downregulation of HIF-1α and Ras [285]. Erianin showed interactions with the binding pockets of HIF-1α and components of the Ras pathway. Synergistic effects resulting from silencing either Ras or HIF-1α, combined with erianin treatment, resulting in reduced PD-L1 expression [246]. These inhibitory effects were mediated by suppressing mTOR/p70S6K/eukaryotic translation initiation factor 4E (eIF4E) and Ras/Raf/MEK/MAPK-ERK signaling pathways [246]. Regulation of tumor angiogenesis by PD-L1 has been previously confirmed alongside its correlation with VEGF expression and MMP-9 inhibition [286], with erianin showing downregulation of both VEGF and MMP-9 expression via PD-L1 silencing [246]. Intercellular adhesion molecule-1 (ICAM-1) and cyclin D1 are also significant regulators of cell proliferation positively correlated with PD-L1 levels [287]. Further treatment with erianin led to the reversal of increased levels of ICAM-1 and cyclin D1 mediated by PD-L1 overexpression. This reversal induced G1 phase arrest, indicating an inhibition in tumor cell proliferation [246]. EMT is an important mediator involved in immune escape, cellular invasion, and migration, which exhibits a strong association with PD-L1, as evidenced by elevated EMT marker expressions upon such upregulation [288]. Notably, erianin treatment subsequently inhibited these effects, leading to suppressed invasion and migration [246].

The growth of tumors was inhibited by either erianin or PD-L1 monotherapy, but no significant synergistic effect was observed. Furthermore, treatment with erianin and combination therapy, in contrast to PD-L1 monotherapy or the control group, improved the immune response by inducing the expression of CD4^+^ and CD8a^+^ T cells in the spleen. Moreover, erianin upregulated *PD-L1* mRNA and protein expression, potentially improving the efficacy of PD-1/PD-L1 antibody treatments. Eriminin is a novel dual inhibitor of MEK1/2 and C-Raf protooncogene serine/threonine protein kinase (CRAF) kinases that inhibits constitutive activation of the MAPK signaling pathway [289].

**Gallic acid**. Gallic acid is a hydroxybenzoic acid derivative commonly distributed across various plant families, such as *Anacardiaceae, Fabaceae*, and *Myrtaceae*, as well as in fungi of the genus *Termitomyces*, vegetable species, and green tea [290] (Fig. 2). It exhibits antioxidant, anti-inflammatory, antibacterial, antiviral, antifungal, antiulcer, and antineoplastic properties [291, 292]. The inhibition of proliferation and induction of apoptosis have been previously reported [290]. In vitro studies indicated a reduction in the expression of *PD-L1* protein and mRNA levels following treatment with gallic acid in a concentration-dependent manner [221]. The impact of p53 on cell cycle arrest and apoptosis induction in response to tumor stresses and its regulation by PI3K/Akt signaling has been established previously [88]. Notably, gallic acid was found to increase p53 protein expression levels in a concentration-dependent manner while downregulating PI3K/Akt phosphorylation [221]. The regulation of PD-L1 expression via p53 is mediated through miR-34a, which is increased after treatment with gallic acid, further indicating its effects on PD-L1 expression [221]. Furthermore, the combinatorial approach utilizing PD-L1 antibody showed an improved therapeutic response characterized by increased PD-L1 expression in tumor cells and improved CD8^+^ T cells effector function [293].

Gallic acid significantly enhanced cytokine production, antitumor effects, and the proliferation of anti-CD19 chimeric antigen receptor T (CAR-T) cells. This enhancement is likely attributed to the stimulation of the IL-4/JAK3-STAT3 signaling pathway. Moreover, gallic acid may specifically target and activate STAT3, thereby contributing to its activation. Combining gallic acid with anti-CD19 CAR-T immunotherapy could be a promising strategy for improving the efficacy of lymphoma treatment [294].

**Hesperidin**. Hesperidin, a flavonoid present in citrus species such as lemons and oranges, has been extensively utilized in traditional medicine and for the treatment of various cardiovascular, neurological, psychiatric, and malignant diseases [295] (Fig. 2). Based on previous studies, hesperidin is known to possess apoptotic-promoting properties and inhibited cell invasion [223]. Flow cytometric analysis of breast cancer cell lines (MDA-MB-231 and MCF) indicated that hesperidin inhibited *PD-L1* mRNA and protein expression in a concentration-dependent manner. Treatment with hesperidin led to the suppression of Akt and NF-κB signaling pathways and the downregulation of PD-L1 expression [223]. The activation of the MAPK/ERK pathway is known to contribute to the immune evasion potential of tumor cells [50], which was further diminished by hesperidin and reduced secretion levels of MMP-9 and MMP-2 in high PD-L1 expressing tumor cells [223]. HN6 and HN15 cell lines showed a decrease in cell viability, colony formation capacity, and migration ability. It also resulted in a decrease in PD-L1 expression levels that were upregulated by IFN-γ treatment. Additionally, hesperidin reduced STAT1 and STAT3 signaling activation in a concentration-dependent manner [296].

Hesperidin inhibits the Akt and NF-κB signaling pathways, delaying the progression of breast cancer, while PD-L1 acts as an upregulator in this process [223]. The rationale for the development of hesperidin as a safer and more potent anticancer agent for NSCLC is based on its ability to inhibit the migratory and invasive capabilities of A549 human NSCLC cells through modulation of the stromal-cell derived factor-1 (SDF-1)/C-X-C motif chemokine receptor 4 (CXCR-4) signaling cascade [297].

**Icaritin**. Icaritin is a hydrolytic product of icariin isolated from traditional Chinese herbal medicine and belongs to the *Epimedium* family [298, 299] (Fig. 2). Icaritin is used to strengthen muscles and bones, kidney detoxification, and immune regulation. It has also been investigated for its anti-inflammatory, antitumor, neuroprotective, and cardioprotective actions [298, 300]. An in vivo study conducted on B16F10 melanoma and the MC38 colon cancer model showed that icaritin treatment increased the number and percentage of CD8^+^ antitumor T cells [224]. Furthermore, Western blotting analysis indicates the inhibiting potential of icaritin on TNF-α and IL-6-induced expression of PD-L1 in neutrophils, with evidence from a combination of icaritin with anti-PD-L1 mAb, indicating effective antitumor immune response [224]. Icaritin blocked the NF-κB signaling pathway by preventing the formation of the IKK complex. This resulted in a concentration-dependent decrease in the nuclear translocation of NF-κB p65 and the downregulation of PD-L1 expression [301].

**Kaempferol**. Kaempferol is a yellow-colored tetrahydroxyflavone compound classified within the aglycone flavonoid group. It is found in various plant parts, including seeds, fruits, and flowers of onion, asparagus, berries, wild leeks, or ramps [302] (Fig. 2). Kaempferol has been widely studied for its numerous health benefits, including anti-inflammatory, antioxidant, antimicrobial, cardioprotective, neuroprotective, hepatoprotective, antidiabetic, and antitumor properties [303, 304]. In a silico study detecting the binding potential of kaempferol, it was observed that kaempferol exhibited binding with the PD-L1 receptor, suggesting a potential interaction [225]. Kaempferol has also been found to inhibit the growth and migration of human CRC and lung metastasis in a concentration-dependent manner. This effect was accompanied by downregulation of metastasis associated lung adenocarcinoma transcript 1 (MALAT1), polypyrimidine tract binding protein 2 (PTBP-2), β-catenin, MMP-7, cellular myelocytomatosis oncogene (c-Myc), and cyclin D1 expression in vitro. Additionally, the growth of orthotopically transplanted tumors was inhibited, leading to prolonged survival rates among tumor-bearing mice [305].

**Licochalcone A**. Licochalcone A is a phenolic chalcone flavonoid derived from the roots of licorice, recognized for its medicinal properties since ancient times [306] (Fig. 2). Various important compounds isolated from licorice, including licochalcone A, exhibit antimicrobial, anticancer, anti-inflammatory, and immunoregulatory activities [306–310]. Inhibition of *PD-L1* protein and mRNA expression was observed in HCT116, HeLa, A549, and Hep3B cell lines without any toxic effects reported [226]. Another study suggested that licochalcone A treatment on cell lines exposed to TNF-α and MG-132 proteasome inhibitor (which prevents PD-L1 degradation) resulted in decreased PD-L1 accumulation. This indicates that licochalcone A affects PD-L1 protein synthesis by suppressing TNF-α induced p-p65 or p65 expressions and nuclear translocation in the colon cancer cell line HCT116. Additionally, inhibition of TNF-α protein, TNF receptor-associated factor-2, and receptor-interacting protein kinase 1 expression, along with IKKα/β phosphorylation has been reported, thus leading to IκB-α degradation and phosphorylation [226]. A molecular docking study demonstrated complete binding between licochalcone A sites and Ras as well as PD-L1 pockets. Immunofluorescence assay in HCT116 cells alongside Western blotting consistently showed a decrease in TNF-α-mediated Ras, p-MEK, and p-Raf expression levels. This collectively resulted in the downregulation of the Ras/Raf/MEK pathway upon treatment with licochalcone A [226]. PARP1 is involved in DNA repair and programmed cell death [311]. Licochalcone A also increased the expression of cleaved PARP and cleaved caspase-8, markers indicative of apoptosis, in HCT116 cells by inhibiting PD-L1 expression.

Xenograft study utilizing the BALB/c male nude mice injected with HCT116 cells also suggested a positive effect of licochalcone A in cancer treatment, as evidenced by a reduction in tumor growth associated with decreased levels of PD-L1, p65, Ras, and VEGF protein in tumor tissues [226]. Licochalcone A-induced suppression of proliferation in PC-3 prostate cancer cell lines in a concentration-dependent manner. This effect was correlated with changes in the expression of DNA polymerase δ and its associated protein, proliferating cell nuclear antigen, leading to the induction of G2/M phase arrest [312]. Furthermore, licochalcone A inhibited the ability of Ras/Raf/MEK and NF-κB signaling pathways to express PD-L1. In a co-culture model of tumor and T cells, licochalcone A suppressed the production of PD-L1, thereby restoring T lymphocyte function [226].

**Luteolin**. Luteolin, a natural flavonoid, is extensively distributed across many plant species, including *Chamaemelum nobile* L., *Petroselinum crispum* (Mill.) Fuss, *Tanacetum vulgare* L., *Trigonella foenum-graecum* L., *Origanum vulgare* L., *Salvia rosmarinus* Spenn*.* (*syn. Rosmarinus officinalis)*, as well as in peppers and carrots [313–315] (Fig. 2). It exhibits a range of potential benefits as an antioxidant, anti-inflammatory, antiallergic, and anticancer agent [314, 316, 317]. In vitro experiments on KRAS-mutated lung cancer cells revealed that luteolin inhibited cell growth and induced apoptosis at minimal concentrations. Additionally, the expression of MUC1-C and PD-L1 was suppressed due to the downregulation of STAT3 phosphorylation following luteolin treatment [227]. Enhanced T cell activity was also observed via increased IL-2 accumulation and inhibition of IFN-γ-mediated interaction between PD-1 and PD-L1 [227].

In vivo studies conducted on H358 and LLC xenograft mouse models using a standard dose of luteolin (30 mg/kg) demonstrated significant inhibition of tumor growth, with tumors exhibiting reduced size, volume, and weight [227]. A further combination approach involving PD-1 mAb and luteolin resulted in improved survival rates, decreased tumor volume and size, diminished lung lesions, increased number of CD8^+^ T cells, and enhanced activation phenotypes [227].

There is growing evidence that PD-L1 expression in lung cancer is upregulated intrinsically through activation of the downstream KRAS signaling pathways [14]. Apigenin has also been shown antitumor properties in genetically modified KRASLA2 mice. In summary, luteolin and apigenin both significantly reduced the growth of lung cancer associated with KRAS mutants and downregulated the expression of PD-L1 induced by IFN-γ. Combining apigenin/luteolin with PD-1 blockade demonstrates a synergistic impact and may be a potential therapeutic strategy for NSCLC with KRAS mutations [227].

**Myricetin**. Myricetin is a flavonoid present in diverse plants, namely *Lycium barbarum* Lam*.*, *Vigna subterranea* (L.) Verdc., *Ribes nigrum* L.*,* *Vaccinium oxycoccos* L., *Empetrum nigrum* subsp*. hermaphroditum* (Hagerup) Böcher, *Capsicum annuum* L., as well as certain vegetables and red wine [318] (Fig. 2). It has a long history of use in traditional Chinese medicine and can be extracted from the bark of the *Myrica rubra* tree [319, 320]. Myricetin exhibits various pharmacological properties, including protective actions on the nervous and cardiovascular systems [321], along with antiviral and immunomodulatory potential [320]. It is also widely studied for its anticancer properties [319]. In vitro flow cytometry analysis indicated that myricetin downregulated IFN-γ-mediated PD-L1 and indoleamine 2,3-dioxygenase 1 expression, resulting in improved T cell activity and enhanced IL-2 secretion. Additionally, Western blotting analysis demonstrated that myricetin inhibited the nuclear accumulation of STAT1, STAT3, and IRF1 in a concentration-dependent manner [229]. IFN-γ stimulated the transcriptional level of PD-L1 via the JAK/STAT/IRF1 axis, which was blocked by myricetin. Collectively, these findings suggest that myricetin holds promise for applications in tumor immunotherapy [229].

**Polydatin***.* Polydatin, a crystalline compound isolated from the roots and rhizome of *Polygonum cuspidatum* Willd. Ex Spreng. [322, 323], is used extensively in Chinese herbal medicine as an analgesic, antipyretic, antiatherosclerotic, and diuretic agent [322] (Fig. 2). It also possesses immune regulatory and antitumor potential [323]. Upon treatment with polydatin, it was observed that the levels of cleaved caspase-3 and cleaved caspase-9 were significantly upregulated, suggesting that polydatin promotes the apoptotic potential of Caco-2 and HCT 116 CRC cell lines in vitro [232, 324]. Polydatin treatment further increased the expression of miR-382, leading to downregulation of PD-L1 levels [103, 232, 325].

An in vivo study conducted on an orthotopic xenograft mouse model using subcutaneously implanted Caco-2 cells demonstrated decreased tumor size and weight, along with upregulation of miR-382 expression after polydatin treatment. The tumor xenograft model showed that polydatin could attenuate tumor growth in CRC [232]. In glioblastoma cells, polydatin enhanced apoptosis while inhibiting cell migration, proliferation, invasion, and stemness properties. Furthermore, it was confirmed that the cytotoxicity of polydatin was associated with the inhibition of the EGFR-Akt/ERK1/2/STAT3-sex-determining region Y-box 2 (SOX2)/Snail signaling pathway, of which several components were essential therapeutic targets for glioblastoma [326].

**Quercetin**. Quercetin, a flavonoid widely distributed in various plants, including *Ginkgo biloba* L., *Hypericum perforatum* L., and *Sambucus canadensis* L. [327, 328], possesses numerous properties such as anti-inflammatory, antioxidant, antiviral, and antitumor potential [327, 329, 330] (Fig. 2). Quercetin has been demonstrated to inhibit the interaction between PD-1 and PD-L1. It also exhibits concentration-dependent inhibition of glycosylated PD-1 and PD-L1 binding, thereby promoting T cell cytotoxicity [233]. Furthermore, quercetin displayed immunoregulatory activities by downregulating NF-κB, ERK, and JNK signaling pathways, subsequently inhibiting the inflammation-producing enzymes such as cyclooxygenase and lipoxygenase [233, 331, 332].

In vivo, studies performed on BALB/c nude mice indicated that quercetin is non-toxic to these animals. The inhibitory effect of quercetin dihydrate on tumor growth is mediated by the reactivation of T cells, as evidenced by increased protein levels of the CD8, granzyme B (GZMB), and IFN-γ in the tumor tissue [233]. Additionally, increased quantities of the CD8^+^ cytotoxic lymphocytes and cytokines, such as GZMB and the IFN-γ, were released from the tumor microenvironment, contributing to tumor cell destruction [233].

The immunomodulatory action of quercetin is mediated through the regulation of the JAK/STAT1 signaling pathway, which facilitates the cooperative destruction of breast cancer cells. By modulating the production of IFN-γ-R, p-JAK2, p-STAT1, and PD-L1 in this pathway while promoting γδ T cell regulation, quercetin acts synergistically to induce apoptosis in breast cancer cells. Consequently, quercetin represents a potential therapeutic agent for adjuvant therapy of breast cancer as well as for the prevention of precancerous conditions associated with this disease [333].

**Resveratrol**. Resveratrol, a phenolic compound initially isolated from *Veratrum grandiflorum* (Maxim. ex Miq.) O. Loes is presented in a variety of foods, including grapes, berries, peanuts, wine, and tea [334, 335] (Fig. 2). It possesses antioxidative, antiaging, and anticancer properties and is recognized for providing numerous cardiovascular benefits [334, 336–338]. In vitro study indicated that thyroxine enhances the effect of PD-L1 on SSC-25 and OEC-M1 oral cancer cell lines when treated with resveratrol. This treatment resulted in the inhibition of PD-L1 protein expression as well as its nuclear accumulation [339]. Furthermore, resveratrol treatment induced conformational changes in PD-L1 structure that increased its electrophoretic mobility while promoting its dimerization and blocking interactions between PD-1 and PD-L1 [340]. Additional in vitro investigations revealed that resveratrol treatment led to upregulation of PD-L1 expression across various tumorigenic cell lines such as BT549, SKBR3, and Cal51 breast cancer cells, BT474 invasive ductal carcinoma, and HCT116, SW480, and SW620 colon cancer cells [234]. The combination of resveratrol with piceatannol, a natural stilbene known for its antitumor properties [341], led to an increase in the expression of γ-H2A histone family member X and cleaved caspase-3. Concurrently, this combinatorial strategy downregulated p38-MAPK and c‑Myc levels, indicative of DNA damage. These findings suggested that resveratrol induces cellular damage while simultaneously elevating PD-L1 expression [234].

The study conducted on BALB/c mice inoculated with 4T1 tumor cells, in conjunction with resveratrol administration, showed reduced lung metastasis, cell proliferation, and inhibition of tumor growth. Resveratrol treatment significantly enhanced the antitumor efficacy of CD8^+^ T cells while further downregulating *PD-1* mRNA expression [342].

The role of IL-6 in the development of cancer-stem-like cells (CSCs) within the niche occupied by oral cancer cells was examined. The mechanism underlying the antitumor action of resveratrol-loaded nanoparticles in oral CSCs was reported to focus on the PD-L1 and IL-6/JAK2/STAT3 signaling cascade. It was determined that the IL-6/PD-L1 axis is essential for CSC proliferation. By suppressing PD-L1 via the IL-6/JAK2/STAT3 pathway, resveratrol-loaded nanoparticles attenuate carcinogenesis in vitro, in ovo, ex vivo, and in vivo model settings. Additionally, this approach disrupts the interaction between AM-M1 macrophages and cancer-associated fibroblasts [343].

**Sativan**. Sativan, a naturally occurring flavone isolated from *Spatholobus suberectus* Dunn, is a Chinese herbal medicine frequently employed for treating various blood disorders [344]. It exhibits anti-inflammatory, antioxidant, and antirheumatic capacities [345] (Fig. 2). Recently, this phytoconstituent has been reported to possess anticancer activity, demonstrating effects against cell proliferation and exhibiting lactate dehydrogenase-inhibitory potential [346]. N-cadherin, Vimentin, and low expression of E-cadherin are considered significant markers for proliferation and PD-L1 expression [347, 348]. Sativan showed inhibition of cell proliferation and viability while enhancing apoptotic potential in a concentration-dependent manner. The suppression of migratory and invasive capabilities was corroborated by polymerase chain reaction analysis, which indicated that sativan increased miR-200c levels in a concentration-dependent manner. This led to the inhibition of the EMT process and PD-L1 simultaneously [236]. In vivo investigations performed on mice xenografted with MDA-MB-232 breast carcinoma cells indicated decreased tumor volume and weight. Immunohistochemical staining revealed the inhibition of PD-L1 expression [236].

**Silibinin**. Silibinin, a phenolic natural bioactive compound extracted from *Silybum marianum* (L.) Gaertn, has been implicated in the treatment of liver diseases [238] as well as various types of tumors, including breast, lung, skin, kidney, colon, and nasopharyngeal cancers [238, 239, 349, 350] (Fig. 2). In several lung cancer cell lines (A549, H292, H460), silibinin induced cell cycle arrest and showed apoptotic potential in a concentration-dependent manner. This effect was further validated by Western blotting analysis comparing the levels of cell cycle marker proteins before and after silibinin treatment [238]. Furthermore, the downregulation of key molecular targets, namely EGFR, STAT5, Akt, MMP-2, MMP-9, and VEGF, along with the inhibition of cellular migration and invasion ability indicated that silibinin reduced angiogenesis, migration, and progression of tumors [238]. Moreover, a significant reduction in p-STAT, p-Akt, and MMP-2 protein levels was observed coupled with inhibition of EGFR-mediated PI3K/Akt and JAK2/STAT5 signaling pathways affecting PD-L1 binding [238]. In the MDA-MB-231 breast cancer cell line model, treatment with silibinin induced apoptosis in a concentration-dependent manner while also promoting mitochondrial fusion among tumor cells, accompanied by decreased mitochondrial reactive oxygen species (ROS) production, which further inhibited cellular migration [350]. In vivo experiments of silibinin on nasopharyngeal cancer models exhibited the suppression of aerobic glycolysis. Flow cytometric analysis confirmed that silibinin interfered with cell survival by inhibiting HIF-1α expression and lactate dehydrogenase A (LDH-A)-dependent glycolytic activity [239].

Silibinin inhibited the expression of phosphorylated EGFR by binding to the receptor, leading to the suppression of downstream targets associated with EGFR, specifically the JAK2/STAT5 and PI3K/Akt pathways. This resulted in decreased expression of PD-L1, VEGF, and MMP. The results of the binding analysis showed that silibinin disrupted the STAT5/PD-L1 complex and demonstrated that STAT5 binds to the PD-L1 promoter region in the nucleus. Collectively, these findings suggest that silibinin may be a potential target for cancer stem cell-directed therapies and tumor immunotherapy [238]. Furthermore, by interfering with HIF-1α/LDH-A-mediated cellular metabolism in nasopharyngeal carcinoma, silibinin can modulate PD-L1 expression [239].

**Shikonin**. Shikonin, a natural naphthoquinone obtained from the roots of *Lithospermum erythrorhizon* Siebold & Zucc.*,* is indicated for various conditions, such as burns, carbuncles, macular eruption, sore throat, and measles [351] (Fig. 2). Several studies have also highlighted its anticancer potential, as this compound induces apoptosis and cell cycle arrest [351–355]. This mechanism by which shikonin exerts these effects involves the generation of ROS and dysregulates intracellular Ca^2+^ levels, leading to its accumulation in mitochondria. The accumulation disrupts microtubule integrity and alters membrane potential [352]. In vitro experiments with shikonin conducted on PC cell lines PANC-1, BxPC3, and 293 T revealed elevated expression of NF-κB, PD-L1, p-STAT3, and CSN5. These findings were also confirmed via the mRNA expression. Western blotting analysis further demonstrated that the inhibition of NF-κB led to a significant reduction in PD-L1 levels. This effect was mediated through the suppression of STAT3 phosphorylation and CSN5 activity. Consequently, shikonin promoted PD-L1 degradation via NF-κB/STAT3 and NF-κB/CSN5 pathways while regulating the PI3K/Akt-mediated signaling in a concentration-dependent manner [237]. Experiments assessing immune evasion status additionally indicated that shikonin increased cleaved caspase-3 levels, suggesting enhanced cytotoxicity against pancreatic tumor cells by PBMCs [237]. Moreover, using male C57B/6J mice with Pan02 cells showed that shikonin facilitated PD-L1 degradation through inhibition of the NF-κB/CSN5 signaling pathway. Different concentrations of shikonin inhibited NF-κB p65 protein expression in a concentration-dependent manner [237].

#### Terpenoids

β-elemene. Elemene is a natural monomeric sesquiterpene compound extracted from the *Curcuma wenyujin* Y.H. Chen & C. Ling [356]. There are three subgroups, namely α-elemene, β-elemene (Fig. 2), and δ-elemene, with β-elemene possessing the highest antitumor activity [241, 357, 358]. Treatment of esophageal cancer cell lines TE-1 and KYSE-150 with β-elemene resulted in a concentration-dependent decrease in cell viability and proliferation. This was accompanied by downregulation of cellular expression of Ki-67, cyclin D1, and cyclin B1, as well as an increase in PTEN expression. These changes led to G-phase arrest and inhibition of proliferation while enhancing apoptotic potential via the cleavage of caspase-3, PARP, and p-Akt. Consequently, this reduced the migration and invasion capabilities of the cancer cells [241]. Additionally, β-elemene inhibited the proliferation of esophageal squamous cell carcinoma by regulating lncRNA-medicated suppression of human telomerase reverse transcriptase expression. This resulted in reduced tumor volume and weight, along with nuclear DNA damage observed in cells treated with β-elemene. These findings indicate that β-elemene promotes apoptosis in esophageal cancer cells [241].

Treatment with β-elemene inhibited the growth of esophageal cancer cells and induced apoptosis. Furthermore, β-elemene effectively prevented the migration and invasion of these cancer cells. Additionally, it selectively modulated Akt signaling, which subsequently regulated PD-L1 expression in esophageal cancer [241].

**Britannin**. Britannin, a sesquiterpene lactone extracted from the perennial plant *Inula britannica* M.Bieb. [359], shows antioncogenic, antidiabetic, antibacterial, and anti-inflammatory properties [360–362] (Fig. 2). Britannin demonstrated a concentration-dependent decrease in *PD-L1* protein and mRNA expression across various cancerous cell lines, including A549, HeLa, Hep3B, HCT116, and human umbilical vein endothelial cells (HUVECs). Flow cytometry confirmed a significant reduction in the population of PD-L1-expressing cells [231]. Molecular docking assays revealed effective binding of britannin to HIF-1α, resulting in downregulation of PD-L1 expression through inhibition of crosstalk between HIF-1α and Myc, further leading to decreased protein expression of Ras, p-Raf, p-MEK, and p-ERK [231]. Treatment with britannin restored and enhanced T cell cytotoxicity while potentially inhibiting the PD-1/PD-L1 interaction. It also contributed to the suppression of tumor angiogenesis as well as reduced expressions of VEGF, and MMP-9 by downregulating PD-L1 expression [231]. The results from an in vivo study on female BALB/c nude mice indicated that britannin significantly inhibited the growth of HCT116 cells along with notable reductions in the tumor size as well as levels of HIF-1α, Myc, PD-L1, and VEGF in tumor tissues [231].

By preventing the formation of the PD-L1 protein without affecting its degradation, bentonina decreased the expression of PD-L1 in tumor cells. Additionally, britannin blocked the ability of the mTOR/p70S6K/4EBP1 pathway to express HIF-1α and inhibited the capacity of Ras/Raf/MEK/ERK pathway to activate Myc. Mechanistically, britannin prevented the interaction between HIF-1α and Myc, thereby suppressing the production of PD-L1 [231].

**Curcumol**. *Curcuma wenyujin* Y.H. Chen & C. Ling*,* a plant belonging to the *Zingiberaceae* family, has been widely used in various regions of the world, particularly in Asian countries such as China, as a traditional herbal medicine [363, 364]. Curcumol (Fig. 2), a sesquiterpenoid and its active ingredient, has been extensively evaluated for its antibacterial, antimicrobial, antitumor, and anticancer properties by researchers [364, 365]. One particular study revealed that curcumol showed inhibitory effects on STAT3 phosphorylation in several cell lines, including A549 lung cancer cells, HeLa cervical cancer cells, and Hep3B liver cancer cells, thus effectively inhibiting tumor progression [244]. The activation of STAT3 is mediated through JAK1 and JAK2 receptor activation [91]. Curcumol was found to suppress the phosphorylation of both receptors. Additionally, Src, a member of the tyrosine kinase family, also contributes to STAT3 activation. Curcumol treatment inhibited constitutive phosphorylation in Hep3B cells [244]. HIF-1α plays a critical role in tumorigenesis through diverse biological processes, such as proliferation, angiogenesis, invasion, metastasis, and cell cycle progression [366]. Evidence suggests that curcumol reduces the regulation of HIF-1α protein synthesis through mTOR/p70S6K/eIF4E and MAPK signaling pathways [244].

In vivo, experiments on a female athymic BALB/c nude mouse model injected with Hep3B cells demonstrated that curcumol significantly reduced tumor size and volume. This reduction was accompanied by decreased protein levels of HIF-1α, PD-L1, VEGF, and p-STAT3 in the tumor tissues. Immunohistochemical analyses revealed that curcumol downregulated the expression of p-STAT3 (T705), HIF-1α, PD-L1, and VEGF in a dose-dependent manner [244]. Additionally, curcumol suppressed PD-L1 expression in hepatic cancer by interfering with the HIF-1α and p-STAT3 (T705) signaling pathways [244].

**Fraxinellone**. Fraxinellone, a natural limonoid compound isolated from the roots and bark of *Dictamnus dasycarpus* Turcz*.,* also known as *Cortex dictamni,* has a long history of use in Chinese herbal medicine [367, 368] (Fig. 2). It has been recognized for its antibacterial, anti-inflammatory, neuroprotective, and anticancer properties [369, 370]. Structurally, fraxinellone is classified as a tetrahydrobenzofuranone derivative and is regarded as a degraded limonoid [371, 372]. Research on various cancerous cell lines, including A549, HeLa, Hep3B, HUVEC, HLF-a, and HCT116, indicated that fraxinellone downregulated PD-L1 expression [248]. Molecular docking analysis confirmed the interaction between fraxinellone and STAT3 at the pTyr705 binding site. In a concentration-dependent manner, fraxinellone inhibited STAT3 transcriptional activity, blocked its phosphorylation and nuclear translocation, and also suppressed the activities of JAK1, JAK2, and Src [248]. The mTOR/p70S6K/eIF4E and MAPK pathways are important mediators of HIF-1α protein synthesis [72, 73], with fraxinellone exhibiting suppression of both pathways, resulting in decreased HIF-1α protein expression [248]. By inhibiting both STAT3 and HIF-1α levels co-operatively leading to reduced PD-L1 protein levels, fraxinellone consequently suppressed cell proliferation [248, 366]. Treatment with fraxinellone resulted in reduced VEGF and MMP-9 levels that were previously enhanced by PD-L1 overexpression, indicating diminished PD-L1 activity [248].

Utilizing an A549 xenograft mouse model, it was observed that fraxinellone effectively suppressed tumor growth. The suppression was accompanied by fluorescent imaging, resulting in decreased expressions of HIF-1α, PD-L1, VEGF, and pTyr705 STAT3 in a dose-dependent manner [248].

**Lycopene**. Lycopene, a tetraterpenoid (carotenoid) derivative, is abundantly present in various fruits and vegetables, particularly in tomatoes, carrots, watermelon, and papaya [373] (Fig. 2). It consists of symmetric hydrocarbon chains with 11 conjugated and 2 non-conjugated bonds. The presence of these conjugated bonds contributes to its strong antioxidant potential [374]. Emerging evidence suggests the significant potential of lycopene in the treatment of various cancer types [375]. In CAL27 and SCC9 cell lines, increased PD-L1 expression was associated with enhanced cell proliferation and inhibition of apoptosis. However, these effects were reversed upon treatment with lycopene, indicating that it inhibits PD-L1 expression. Additionally, lycopene treatment reduced the levels of type 1 insulin-like growth factor receptor (IGF-1R), p-Akt1, NF-κB, STAT3, and c-Myc, further suggesting a reversal of actions linked to PD-L1 overexpression [228]. It was reported that lycopene enhances the efficacy of anti-PD-1 therapy via activating IFN-γ signaling in lung cancer cells [252]. Lycopene interacted with IL-1 and IFN-γ to reduce levels of IL-4 and IL-10 in the spleen of mice. This interaction also influenced the CD4^+^/CD8^+^ ratio in the spleen while promoting IFN-γ-expressing CD8^+^ T cells in tumor tissues [252]. Combinatorial treatment resulted in enhanced cell apoptosis alongside significantly upregulated mRNA levels of *IFN-β*, *IFN-γ*, *IRF1*, *IRF7*, *CXCL9*, and *CXCL10* as well as decreased the methylation levels of IRF1 and IRF7 [252].

**Triptolide**. Triptolide belongs to the triterpene epoxide family. It is well-known in traditional Chinese medicine as *Tripterygium wilfordii* Hook. F. [376] (Fig. 2). Triptolide exhibits a variety of effects, including anti-inflammatory, antioxidant, immunomodulation, neuroprotection, and anticancer activities [377, 378]. In an in vitro oral cancer model, PD-L1 expression was significantly upregulated; however, this expression markedly decreased upon triptolide treatment, which further inhibited tumor growth, confirming that this inhibition was mediated through downregulation of JAK/STAT1 and HER2/phosphoinositide 3-kinase/Akt signaling pathways [239]. Various experiments conducted on glioma cell lines indicated that PD-L1 expression induced by IFN‑γ was reduced. Additionally, previous reports noted a reversal of T cell and CD4^+^ T cell inhibition alongside decreased levels of IL-2 and IL-10 secretion following treatment with triptolide [379].

In vivo experiments using different xenograft models derived from patient tumors, including oral squamous cell carcinoma and oral SAS cells, also demonstrate reduced PD-L1 expression, decreased tumor growth, and diminished cell proliferation [240].

**Ginsenoside Rk1**. Ginsenoside Rk1, a saponin component derived from *Panax ginseng* C.A.Mey. (ginseng), has been traditionally used in herbal medicine [380, 381] and exhibits diverse effects such as anti-inflammatory [381] and anticancer potential [382] (Fig. 2). It has also been associated with the regulation of endothelial functions [383, 384]. In vitro studies indicated that ginsenoside Rk1 decreased the proliferation of lung adenocarcinoma cells and induced G-phase arrest, accompanied by upregulation of Bax, cleaved caspase-3, caspase-8, and caspase-9 as well as PARP expression while downregulating Bcl-2 expression, indicating a caspase-dependent apoptotic mechanism [222]. NF-κB-mediated regulation of PD-L1 expression has been studied previously in vitro, suggesting that ginsenoside Rk1 downregulated NF-κB activity, further leading to inhibition of PD-L1 expression [222].

**Panaxadiol**. Panaxadiol, a terpenoidal saponin derived from the roots of *Panax ginseng* [385], has been used in traditional medicine for its anti-inflammatory, anticancer, and neuroprotective potential [386, 387] (Fig. 2). In vitro analysis on various colon cancer cell lines such as HCT116, SW620, and HT-29, indicated that panaxadiol treatment resulted in decreased *PD-L1* protein and mRNA levels alongside a reduction in HIF-1α overexpression, as confirmed by Western blotting and immunofluorescence staining [230]. Treatment with panaxadiol reduced PD-L1 expression induced by STAT3 activation while also suppressing JAK1, JAK2, and Src expression in a concentration-dependent manner. This was associated with enhanced T cell cytotoxicity and the inhibition of the interaction between PD-1 and PD-L1 [230].

In a vivo xenograft model utilizing HCT116 cells, panaxadiol demonstrated suppression of tumor growth without notable changes in body weight. It also led to decreased levels of HIF-1α, p-STAT-3, PD-L1, and VEGF in a dose-dependent manner, consistent with the previous in vitro findings [230].

### Marine natural compounds

#### Fucoidan

**Fig. 3:**
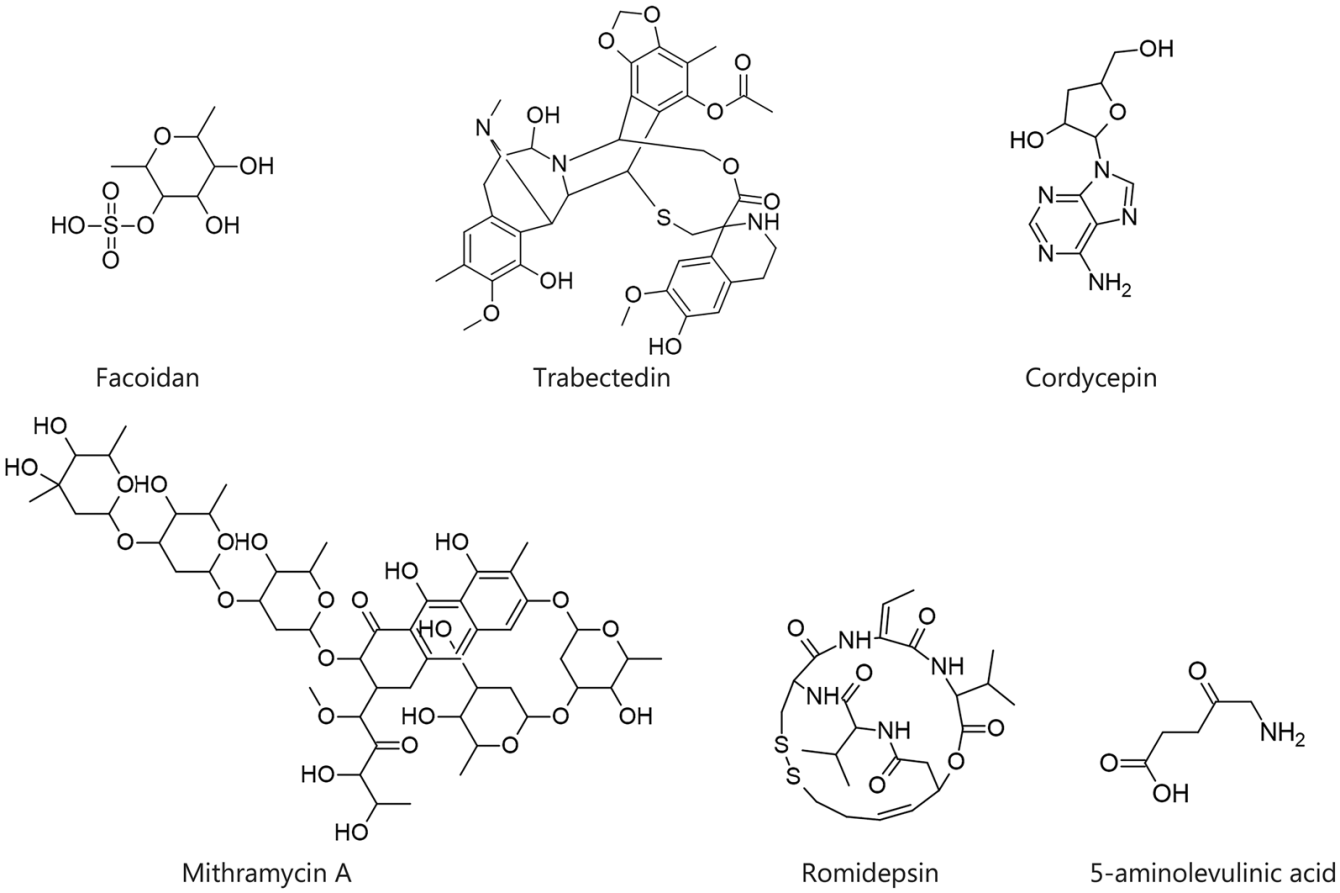
Chemical structures of bioactive anticancer natural compounds from non-plant sources targeting PD-1/PD-L1

Fucoidan is a fucose-rich polysaccharide that is abundant in brown seaweeds and algal species, such as *Fucus evanescens*, several species of *Laminaria*, and land plants like *Ferula hermonis* [388–390] (Fig. 3). Fucoidan-rich algal supplements are marketed for nutraceutical [388], therapeutic [391], cosmetic [392], and diagnostic purposes [393]. It has demonstrated efficacy in reducing the growth of diverse cancers, including ER-dependent and -independent breast cancer cell lines, PC-3 prostate cancer cells, SNU-761 HCC cells, and lung cancer in Lewis tumor-bearing mice [394, 395]. Experiments conducted on HT1080 fibrosarcoma cell lines revealed suppressed mRNA levels of *PD-L1*, *EGFR*, and *VEGF* without affecting the binding between PD-1 and PD-L1 upon treatment with low molecular weight fucoidan [396].

In vivo experiments investigating breast tumor development induced by dimethylbenz(a)anthracene (DMBA) injection indicated decreased tumor growth in fucoidan-treated animals. These animals showed a higher percentage of TCRαβ^+^CD161a^+^ NK cells along with increased CD4^+^ and CD8^+^ T cells. Furthermore, enhanced levels of IL-6, IL-12, p40, and IFN-γ were observed alongside decreased levels of Foxp3, p-PI3K, p-Akt, and PD-L1 following fucoidan treatment [251].

#### Trabectedin

Trabectedin, also referred to as yondelis, is a tetrahydroisoquinoline alkaloid originally extracted from the marine organism *Ecteinascidia turbinate*. It has demonstrated promising antitumor activity against breast cancer, oral cancer, renal cancer, prostate cancer, and NSCLC [397–400] (Fig. 3). The combination of trabectedin treatment with α-PD-1 mAb led to decreased cell growth of ID8 cells in vitro. Further exploration indicated enhanced survival rates and reduced tumor growth, coupled with an increased number of IFN-γ-induced CD4^+^ and CD8^+^ effector T cells alongside decreased number of Tregs and MDSCs [254].

In vivo studies conducted on patient-derived chronic lymphocytic leukemia xenografts indicated enhanced antitumor activity. Treatment with trabectedin resulted in the depletion of Ly6C monocytes expressing PD-L1, a reduction in the expression of *Foxp3* mRNA, and a decrease in immunosuppressive Tregs. Furthermore, trabectedin interfered with the PD-1/PD-L1 axis, leading to diminished levels of CD8^+^ T cells expressing PD-1 [401]. The rapid activation of caspase-8 via membrane signaling through tumor necrosis factor-related apoptosis-inducing ligand (TRAIL) receptors, expressed at higher levels in monocytes and macrophages compared to neutrophils and lymphocytes, has been associated with the exceptional selectivity of trabectedin for mononuclear phagocytes (monocytes and macrophages) [402].

### Microbial agents

#### Cordycepin

Cordycepin, isolated from the caterpillar fungus *Cordyceps militaris*, is an adenosine analog that contains multiple bioactive compounds [403, 404] (Fig. 3). It is well known for its therapeutic activities, such as anti-inflammatory, antiangiogenic, antitumor, immunomodulatory, and antidiabetic effects [405–409]. In vitro studies on 4NAOC-1 oral cancer cells treated with cordycepin and its preparation indicated inhibition of cell proliferation, increased cell apoptosis, decreased G2/M phase cell cycle progression, and reduced expression of p-EGFR, p-ERK1/2, p-STAT3, p-p70S6K, β-catenin, IL-17RA, and cyclin B1. Additionally, there was an increase in the expression of p-STAT1, leading to downregulation of PD-L1 but not PD-L2 in vitro. Further treatment with cordycepin in a mouse tumor model revealed reduced expression of Ki-67, p-EGFR, and p-STAT3 in cancer tissues [243]. Investigations suggest that cordycepin may target and disrupt the activity of various kinases, including but not limited to JNK, MAPK, adenosine monophosphate-activated protein kinase (AMPK), PI3K/Akt, ERK, GSK-3β, and focal adhesion kinase pathways [410].

#### Mithramycin A

Mithramycin A is a polyketide antibiotic produced by the soil bacterium *Streptomyces*, known to bind to the minor groove of DNA and inhibit the binding of the transcription factor specificity protein 1 (SP1) to DNA [411, 412] (Fig. 3). Mithramycin A has been found to possess calcium-lowering and antitumor properties as well [411, 413, 414]. An in vivo study conducted on orthotopic tumor-bearing C57BL/6 mice injected with MC38 cells indicated enhanced CD8^+^ T cell infiltration in tumors, thereby arresting tumor growth when combined with mithramycin A and α-PD-L1 blocker. When administered as monotherapy in vitro, mithramycin A enhanced the levels of PD-L1 in a concentration-dependent manner, which was further confirmed in vivo using CD45^−^ tumor-bearing mice. Furthermore, treatment with mithramycin A alone and in combination with PD-1 therapy resulted in a decrease in the *CCL2* gene expression in vivo and a reduction of M2 macrophages (CD206^+^F4/80^+^) after mithramycin A and α-PD-L1 treatment [253].

Compared to mithramycin A and anti-PD-1 antibody administered alone, the combination of these two agents showed enhanced anticancer activity in MC38 allograft mice. Mithramycin A, when evaluated using an immune checkpoint array, decreased the expression of Fas ligand and galectin-3. The results also showed that mithramycin A downregulates CD47 expression while upregulating PD-L1 expression. Additionally, the combination of anti-PD-1 antibody and mithramycin A exhibits a potent anticancer effect [415]. Mithramycin A suppressed the growth of cervical cancer by disrupting the death receptor 5/caspase-8/Bid signaling pathway, resulting in proteasome-dependent degradation of Sp1 [416].

#### Romidepsin

Romidepsin, a naturally occurring compound isolated from the Gram-negative soil bacterium *Chromobacterium violaceum* [417, 418], is a selective inhibitor of the HDAC1 and HDAC2, which are enzymes involved in the regulation of gene expression [419, 420] (Fig. 3). It has been reported that romidepsin induces cell cycle arrest and apoptosis in various solid tumor cells, including ovarian cancer and HCC [420, 421]. In addition to its direct cytotoxicity, romidepsin can elicit a wide range of immune changes similar to those observed with other HDAC inhibitors. This includes the upregulation of costimulatory molecules, such as PD-L1, MHC, tumor antigens, and cytokines. Evidence suggests an increase in Foxp3 expression and suppressive activity of Tregs in inflammatory and transplantable tumor models [422, 423]. Treatment with romidepsin regulated cell proliferation, differentiation, and apoptosis, leading to PD-L1 upregulation in CLC cells. This upregulation correlated with increased levels of histone H3/H4 acetylation and regulation of BRD4 in CT26 and MC38 cell lines. The enhanced PD-L1 expression was further associated with increased T cell infiltration, downregulation of IFN-γ and IL-4, along with the secretion of IFN-γ in CD8^+^ T cells, as well as upregulation of Foxp3^+^ Tregs [235].

Romidepsin treatment inhibited tumor growth despite its series of immunosuppressive effects, as demonstrated by the findings from colitis-associated cancer models and subcutaneous tumor-transplanted animals [235]. Consequently, romidepsin plus anti-PD-1 antibody offers a more promising therapeutic approach for colon cancer. Mechanistically, tyrosine phosphatases are recruited to dephosphorylate downstream effector molecules, thereby attenuating TCR-mediated signaling, which in turn prevents T cell proliferation and cytokine production [424, 425]. This process occurs after the interaction between the PD-1 receptor on T cells and its ligand PD-L1. Antibodies that block PD-1 can impede the PD-L1/PD-1 pathway and restore T cell activity. Conversely, T cell responses are adversely regulated by PD-L1 expression on tumor cells, allowing for immune escape [235, 426, 427].

### Miscellaneous compounds

5-aminolevulinic acid is a naturally occurring amino acid synthesized in all nucleated mammalian cells and plays a crucial role in various biochemical pathways for the formation of essential photosensitive components [428] (Fig. 3). Currently, 5-aminolevulinic acid and its derivatives have been approved by the FDA for the treatment of actinic keratosis, basal cell carcinoma, and tumor diagnosis [428]. Moreover, 5-aminolevulinic acid has been approved for treating acne vulgaris in China [429].

In vivo experiments conducted on B6 mice demonstrated a potent tumor inhibitory effect when combining 5-aminolevulinic acid with anti-PD-L1 mAb, yielding similar results in B16BL6 and C26 models alongside an increase in the T cell population [249]. The experimental model also showed enhanced mitochondrial function through the activation of peroxisome proliferator-activated receptor-gamma coactivator (PGC)-1α (PGC-1α), leading to improved PD-1/PD-L1 blockade [249].

## Clinical studies

Clinical studies that involve natural compounds targeting PD-1/PD-L1 are essential for developing more effective and less toxic cancer treatments. Natural compounds represent a substantial yet largely mainly unexplored reservoir of bioactive molecules with potential anticancer activities. Through the clinical evaluation of these natural agents, novel PD-1/PD-L1 inhibitors could improve or complement existing therapies, offering patients a wider range of therapeutic options. Additionally, by establishing the safety, efficacy, and optimal application of natural agents in oncology, these trials may pave the way for less harmful and more effective breakthroughs in cancer immunotherapy compared to traditional treatments.

A critical analysis of natural agents studied in vitro or in vivo for targeting PD-1/PD-L1 reveals that numerous natural compounds demonstrated efficacy against cancer across various studies, with many either having undergone clinical evaluation or currently being evaluated. Numerous clinical trials are being performed with natural compounds, such as berberine, EGCG, icaritin, quercetin, resveratrol, silibinin, lycopene, ginsenoside Rg3, fucoidan, and cordycepin. Additionally, several other natural compounds are also in early-phase clinical trials. The detailed summary of these trials is presented in Table 5 [430–438]. Most clinical trials were at phase I or phase II stages, with some progressing to phase III. Notably, many of these trials involved a small number of participants, which is a significant limitation that can be addressed in the future. Some studies are currently at the dose escalation stage for agents, such as cordycepin and mithramycin (NCT00003005, NCT01610570, and NCT01317953). Furthermore, a few trials were terminated due to severe/serious adverse events or other reasons (NCT01610570, NCT03236649, NCT00920556, and NCT00920556). Specific patterns were also observed, for instance, many clinical studies involving EGCG focused on HPV treatment. Similarly, tumor growth may be inhibited by *Veillonella parvula* through the reduction of B cells immunomodulatory activity mediated by berberine [430, 431]. Lycopene has primarily been evaluated for prostate cancer treatment. Apart from the aforementioned studies, several ongoing investigations with recruiting status involve icaritin. Multiple clinical trials have been conducted with quercetin, mostly associated with chemoprevention and its combination therapies aimed at overcoming chemoresistance. Certain formulations of quercetin and resveratrol, including nano- and micro-formulations, have undergone clinical evaluation. Pharmacokinetic studies have also been performed to assess the bioavailability and other parameters related to these natural compounds (NCT00920803).

**Table 5:** Overview of various concluded or ongoing clinical trials for various natural compounds

Intervention	Phase	Enrollment (actual or estimated)	Condition	Dose and duration	Country	Main findings	Reference
Berberine hydrochloride	Phase II and III	1000	Colorectal adenoma (in patients with previous colorectal cancer)	300 mg tablet orally, twice daily for 3 years	China	Not reported	NCT03281096
	Phase II and III	100	Colorectal adenoma	100 mg tablet orally, twice daily for 6 months	China	Not reported	NCT03333265
	Phase II and III	1108	Colorectal adenoma	0.3 g twice per day for 2–3 years	China	Berberine hydrochloride was safe and effective in lowering recurrence of colorectal adenoma	NCT02226185 [430, 431]
Berberine chloride	Phase I	18	Colorectal cancer, ulcerative colitis	Dose not reported, TID, *p.o.* for 90 days	China, USA	A mild adverse event without any serious adverse events	NCT02365480
Dietary supplements containing apigenin and EGCG	Phase II	382	Colorectal cancer	Flavonoid mixture (tablet) with apigenin (20 mg) and EGCG (20 mg) per day	Germany	Trial suspended	NCT00609310
Cinnamon	Early phase I	16	Drug-food interactions	2 g (oral capsule)	USA	Significant interactions with nicotine with prolonged cinnamon exposure	NCT05157672 [432]
EGCG	Early phase I	51	Colon Cancer	94% EGCG (450 mg) *p.o.* twice daily	USA	Not reported	NCT02891538
	Phase I	Recruiting	Small cell lung carcinoma	Dose escalation (400, 800, 1200, 1600, 1600, and 2000 mg, BID)	China	Recruiting	NCT01317953
	Phase II	72	Esophageal squamous cancer	EGCG solution (4400 µmol/L)	China	Not reported	NCT06398405
	Phase I	18	Esophageal cancer	Repeated swallowing of 30 ml of the EGCG solution (880, 1760, 2640, 3430, and 4400 µmol/L per dose)	China	Not reported	NCT05039983
Icaritin	Phase Ib	28	Advanced solid tumors	600 mg, 800 mg two doses, BID, continuous dosing for 56 days	China	Early benefits for durable survival in patients with advanced HCC	NCT02496949
	Phase I	30	Advanced breast cancer	50, 100, 200, 300, 400, and 500 mg ascending multiple oral doses, QD; single and continuous dose for 28 days	China	Not reported	NCT01278810
	Phase II	70	Advanced HCC	600 mg orally, twice daily for a total daily dose of 1200 mg	China	Not reported	NCT01972672
	Phase III	89	Advanced HCC	600 mg/time twice daily (30 min after breakfast, lunch, and dinner), *p.o*	China	Terminated due to change in clinical development plan	NCT03236649
Licochalcone A	Phase I	18	Oral squamous cell carcinoma	Not available	Egypt	Not reported	NCT03292822
Quercetin with or without nanoparticle	Early phase I	200	Metastatic breast cancer and triple-negative breast cancer	Combination of quercetin, EGCG, metformin and zinc	Saudi Arabia	Not reported	NCT05680662
	Phase II	1,000,000	Squamous cell carcinoma	Quercetin and its encapsulated PLGA-PEG nanoparticles	Egypt	Not reported	NCT05456022
	Not applicable	60	Prostate cancer	500 mg/d quercetin along with some other vitamins for 6 months	Germany	Not reported	NCT01538316
	Phase I	32	Prostate cancer	Quercetin given *p.o.* BID for 3–6 weeks	USA	Not reported	NCT01912820
Genistein	Not applicable	60	Prostate cancer	100 mg/days quercetin along with some other vitamins for 6 months	Germany	Not reported	NCT01538316
Tislelizumab + dasatinib + quercetin (neoadjuvant)	Phase II	24	Head and neck squamous cell carcinomas	Quercetin (1250 mg/days)	China	Not reported	NCT05724329
Micronized resveratrol (SRT501)	Phase I	9	Colorectal cancer and hepatic metastases	5 g *p.o.*, once daily for 14 days	USA and UK	The intervention was well tolerated with higher C_max_ of the micronized formulation than normal with a significant effect against colorectal cancer	NCT00920803 [433]
Resveratrol with or without grape powder	Not applicable	07	Low-grade gastrointestinal tumors	5 g/d p.o., in two divided doses of 2.5 g each without a break in therapy for a total of 3 cycles	USA	Not reported	NCT01476592
	Phase I	11	Colon cancer	Resveratrol tablets at 20 and 80 mg/days, grape powder at a dose of 80 and 120 g/days orally	USA	The highest effect was reported from grape powder that could block the Wnt pathway in vivo and this impact is limited to the normal colonic mucosa resveratrol also showed a limited effect	NCT00256334
	Phase I	40	Healthy volunteers	0.5, 1.0, 2.5, or 5.0 g/days for 29 days	UK	Caused mild to moderate gastrointestinal effects (2.5 and 5 g), but the supplement was safe. Reduced blood IGF-I and IGFBP-3 levels	NCT00098969
SRT501 with or without bortezomib	Phase II	24	Multiple myeloma	SRT501 5.0 g	UK and USA	Terminated due to serious adverse events. The adverse effects that were recorded with anaemia, vomiting, diarrhea, fatigue, and nausea as most frequent. Two fatalities (one likely connected to medication)	NCT00920556 [434]
Silibinin	Not applicable	70	Brain metastases and breast cancer	1 g/days (*p.o.*) plus standard systemic therapy for NSCLC or breast cancer	Italy	Not reported	NCT05689619
Silibin-phytosome	Phase II	12	Prostate cancer	13 g daily dose for 2–10 weeks, in three divided doses	USA	While high-dose oral silybin-phytosome temporarily raised blood concentrations, prostate tissue exhibited low silibinin levels. After surgery, one of the treated patients experienced a grade 4 thromboembolic episode. One patient with asymptomatic grade 2 hyperbilirubinemia and four with diarrhea	NCT00487721 [435]
β-Elemene	Phase III	100	Malignant gliomas	600 mg/days, i.v., day 1–14, every 28 days for 1 cycle, for 6 cycles	China	Not reported	NCT02629757
Lycopene with or without docetaxel	Phase II	66	Intraepithelial prostatic neoplasia, prostatic neoplasms	30 mg/days	USA	No serious adverse events	NCT01443026
	Phase II	47	Prostate cancer	15 mg twice daily *p.o*	USA	Significant decline in serum PSA level in one patient with certain adverse effects. Not very effective for androgen-independent prostate cancer	NCT00068731
	Phase I	Not provided	Prostate cancer	Lycopene single dose orally (a mixture of tomato paste, water, and olive oil)	USA	Not reported	NCT00006078
	Phase I	Not provided	Prostate cancer	Lycopene capsules single dose (dose escalation study)	USA	Not reported	NCT00093561
	Phase II	14	Prostate cancer	Docetaxel (75 mg/m^2^) and lycopene (30 mg) daily for every 21 days for at least 4 courses	USA	Improved PSA response rates compared to single-agent docetaxel	NCT01882985 [436]
	Not applicable	84	Prostate cancer	Two 15 mg lycopene capsules daily for 3 months	USA	No specific genes have been definitively identified as being directly associated with dietary fish oil or lycopene consumption	NCT00402285
	Phase II	10	Prostate cancer	Lycopene for (6 ± 1) weeks of preoperative supplementation with 60 and 30 mg/days lycopene QD for 4–7 weeks	USA	Only the primary measures were examined as the trial closed before the accrual goals were met	NCT00450749
Ginsenoside Rg3	Phase II	480	HCC	2 capsules 20 mg, BID, 8 weeks as one cycle	China	Not reported	NCT01717066
	Phase II	100	Advanced gastric cancer	Ginsenoside Rg3 20 mg 2/days *p.o.* and first-line chemotherapy	China	Not reported	NCT01757366
Red ginseng	Not applicable	60	Gastrointestinal cancer	Red ginseng tablet 500 mg/T for 3 months after surgery	Republic of Korea	Not reported	NCT06561516
Fucoidan	Not applicable	100	Cancer cachexia and sarcopenia	Dose not reported, 2 servings per day, a total of 8 tablets one time or in a divided dose	Taiwan (Republic of China)	Not reported	NCT05623852
	Not applicable	100	Advanced HCC	4.4 g oligo fucoidan powder, *p.o.*, BID	China	Suspended	NCT04066660
	Not applicable	60	Metastatic colorectal cancer	4 g, *p.o.* BID for 6 months	Taiwan (Republic of China)	The disease control rate was greatly enhanced by the combination of chemotarget drugs and fucoidan	[437]
Trabectedin	Phase II	271	Advanced cancer	1.5 mg/m^2^ as 24 h i.v. infusion on day 1 of every 21-day cycle; 0.58 mg/m^2^ as a 3 h i.v. infusion on days 1, 8, and 15 of each 28-day cycle	USA	The first dose regimen produced a longer time to progression and progression-free survival	NCT00060944 [438]
Cordycepin Cordycepin + pentostatin	Phase I	14	Leukemia	Dose escalation: 6, 12, 24 and 48 mg/m^2^	USA	Not reported	NCT00003005
	Phase I and II	44	Refractory TdT-positive leukemia	Dose not reported, days 1, 2, and 3 of a 21-day cycle	USA	Not reported	NCT00709215
Mithramycin	Phase I and II	8	Solid tumors or Ewing sarcoma	9.0 mcg/kg; 13.0, 17.5, and 17.5 mcg/kg (dose escalation study)	USA	Terminated; no participants were enrolled on additional dose levels because the study’s enrolment period ended before the dose level 1 was finished	NCT01610570

*BID* bis in die (twice daily), *C*_*ma*x_ maximum plasma concentration, *EGCG* epigallocatechin gallate, *HC*C hepatocellular carcinoma, *IGF-I* insulin-like growth factor, *IGFBP* insulin-like growth factor binding protein, *mg p.o., per os*, *NSCLC* non-small cell lung cancer, *PLGA-PEG* poly(lactide-co-glycolide)-poly(ethylene glycol), *PSA* prostate-specific antigen, *TdT* terminal deoxynucleotidyl transferase, *TID* ter in die (3 times a day), *QD* quater die sumendus (4 times a day), *V. parvula Veillonella parvula*

In a limited number of studies, serious adverse events were reported (NCT00920556), likely attributable to the dosage and tolerance of the natural compound evaluated. Previous reports indicate that many natural compounds, particularly those with anticancer properties, can lead to significant adverse effects [439]. This underscores the necessity for careful consideration in optimizing and finalizing dosages. Regarding the comparison of safety and toxicity profiles among natural compounds, some, such as romidepsin, are already available on the market as anticancer drugs. While it is generally perceived that natural compounds are relatively safer than their synthetic counterparts, it is important to note that in many clinical studies, these compounds are utilized primarily as adjuvants or supplements. Additionally, several trials have been terminated due to serious adverse events; hence, establishing the safety and appropriate dosing of natural compounds remains imperative.

## Conclusions

Growing research in the field of immune checkpoint blockade therapy has provided a guided approach to treating various tumors. An increasing emphasis on exploring newer naturally derived compounds has led to enhanced investigations in this area, further broadening the potential for developing innovative therapeutic strategies. Additional research is necessary to fully harness the benefits of these bioactive compounds in the treatment of tumor and disease progression, as well as to promote strategic guidelines for developing new natural product-based therapies.

**Fig. 4:**
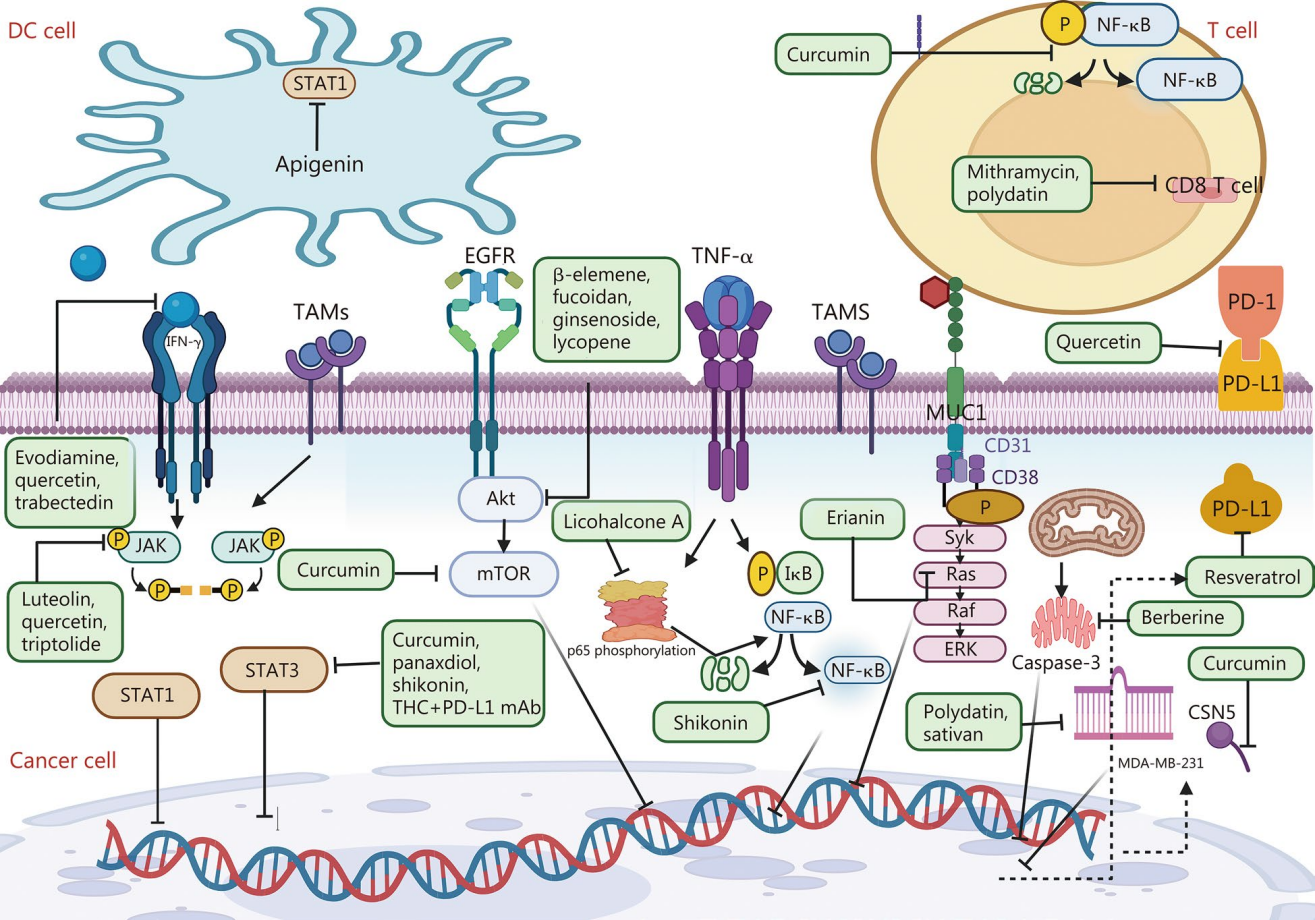
Mechanistic overview of targeting PD-1/PD-L1 by bioactive natural compounds. Various natural compounds inhibit signaling pathways involving JAK-STAT and Ras/Raf. Berberine, for example, decreases PD-L1 and Ki-67 levels while increasing cleaved caspase-3. Apigenin and luteolin inhibit IFN-γ-induced PD-L1 expression and STAT3 phosphorylation. Curcumol reduces HIF-1α and VEGF protein levels, limiting crosstalk between STAT3 and HIF-1α pathways and restricting tumor growth. Evodiamine significantly reduces MUC1-C expression, modulating tumor development. Licochalcone A negatively regulates NF-κB and Ras/Raf/MEK signaling pathways. Erianin regulates Ras, VEGF, and HIF-1α expression, inhibiting tumoral growth. β-elemene inhibits p-Akt expression, while lycopene reduces IL-4 and IL-10 levels, enhancing antitumoral T cell activity. Panaxadiol restores T-lymphocytes’ tumor-killing activity, and sativan and polydatin regulate miRNA levels, controlling tumoral cell cycle pathways. Shikonin leads to CSN5 inactivation, degrading PD-L1, and activating tumor infiltration by T cells. HIF-1α hypoxia-inducible factor 1-α, EGFR epidermal growth factor receptor, ERK extracellular signal-related kinases, IFN-γ interferon-γ, JAK Janus kinase, MUC1 mucin 1, mTOR mammalian target of rapamycin, NF-κB nuclear factor-κB, PD-1 programmed cell death protein 1, PD-L1 programmed cell death ligand 1, Ras rat sarcoma virus, Raf rapidly accelerated fibrosarcoma, STAT signal transducer and activator of transcription, Syk spleen associated tyrosine kinase, TAMs tumor-associated macrophages, TNF-α tumor necrosis factor-α, THC ( −)-trans-∆9-tetrahydrocannabinol, IκB inhibitor of nuclear factor-κB, CD cluster of differentiation, CSN5 constitutive photomorphogenic-9 signalosome 5, COP9 signalosome subunit 5

**Fig. 5:**
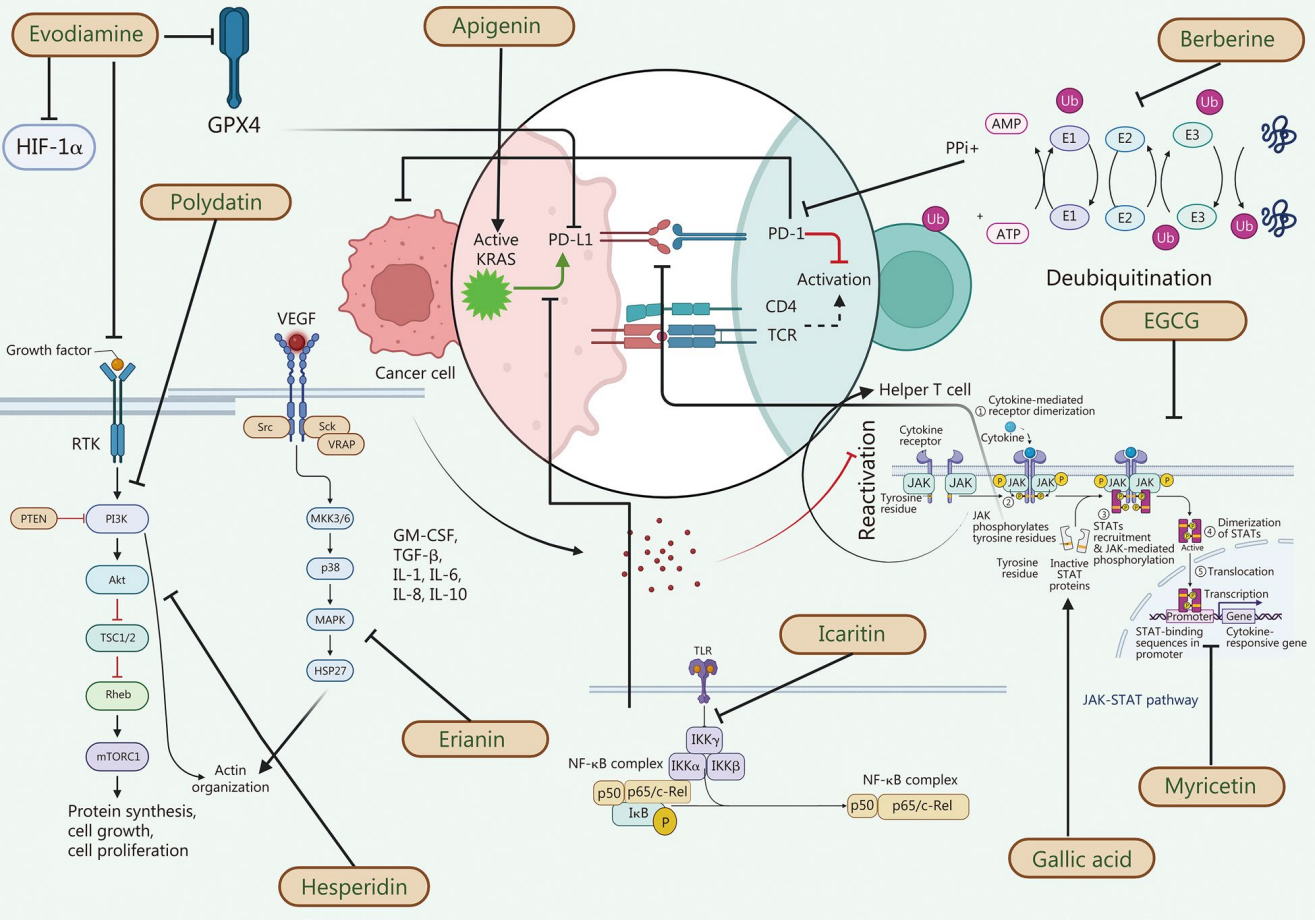
Impact of natural compounds targeting PD-1/PD-L1 on various signaling pathways. AMP adenosine monophosphate, ATP adenosine triphosphate, ERK extracellular signal-related kinases, HIF-1α hypoxia-inducible factor 1-α, IFN-γ interferon-γ, JAK Janus kinase, KRAS Kirsten rat sarcoma viral oncogene homologue, mTOR mammalian target of rapamycin, NF-κB nuclear factor-κB, PD-1 programmed cell death protein 1, PD-L1 programmed cell death ligand 1, RTK receptor tyrosine kinase, STAT signal transducer and activator of transcription, Syk spleen associated tyrosine kinase, TLR Toll-like receptors, GPX glutathione peroxidase, PTEN phosphatase and TENsin homolog deleted on chromosome 10, PI3K phosphoinositide 3-kinase, TSC tuberous sclerosis complex, MKK mitogen-activated protein kinase, MAPK mitogen-activated protein kinase, HSP27 heat shock protein 27, GM-CSF granulocyte–macrophage colony-stimulating factor, TGF transforming growth factor, IL interleukin, CD cluster of differentiation, TCR T cell receptor, PPi protein protein interactions, E enzyme, EGCG epigallocatechin gallate, IκB inhibitor of nuclear factor-κB, IKK inhibitor of nuclear factor-κB kinase, Rheb Ras homolog enriched in brain, VEGF vascular endothelial growth factor, Src Src kinase, Sck C-terminal Src kinase, VRAP VEGF receptor associated protein

**Fig. 6:**
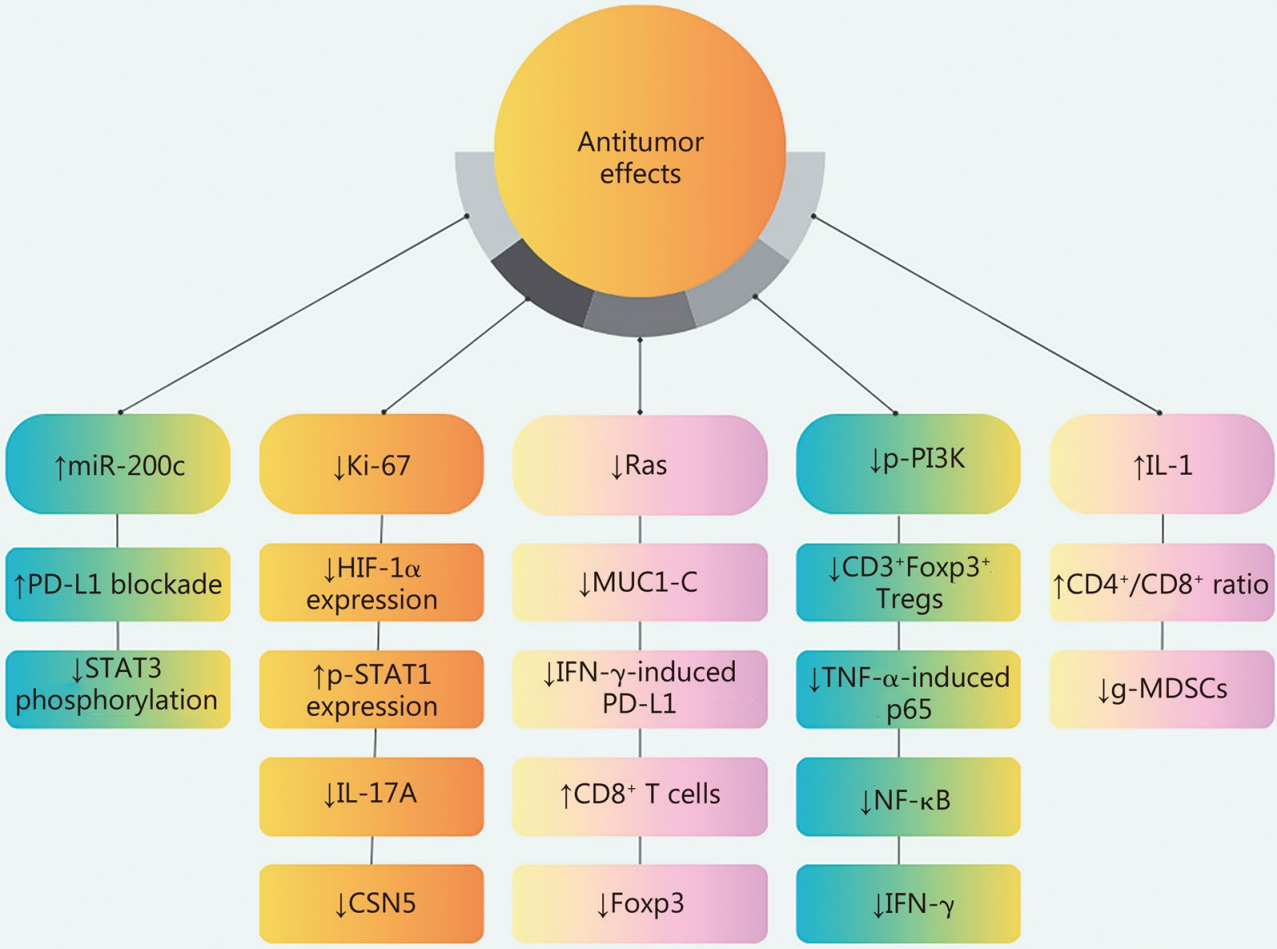
An overview of various mechanisms involved in the antitumor effects of natural compounds targeting PD-1/PD-L1. PD-1 programmed cell death protein 1, PD-L1 programmed cell death ligand 1, miR-200c microRNA 200c, STAT signal transducer and activator of transcription, IL interleukin, CSN5 constitutive photomorphogenic-9 signalosome 5, Ki-67 antigen Kiel 67, Ras rat sarcoma virus, MUC1-C mucin 1 C-terminal subunit, IFN-γ interferon-γ, CD8^+^ cytotoxic T cells, PI3K phosphatidylinositol 3-kinase, Foxp3 forkhead box P3, TNF-α tumor necrosis factor-α, NF-κB nuclear factor-κB, MDSCs myeloid-derived suppressor cells

Recent research has revealed that natural compounds are effective against several cancer types. Many natural compounds have been developed into approved anticancer drugs, while others are under intense investigation. The role of these natural compounds in the PD-1/PD-L1 axis, which is central to cancer immunotherapy, is expected to be used in combination with existing antibody therapies. Therefore, it is hoped that the systematically summarized data present in this review will facilitate the development of clinical application research in the future. As illustrated in in Figs. 4–5, various natural compounds inhibit signaling within JAK/STAT and Ras/Raf-mediated pathways. Berberine decreases PD-L1 and Ki-67 levels while increasing levels of cleaved caspase-3, apigenin, and luteolin, leading to inhibition of IFN-γ-induced PD-L1 expression and STAT3 phosphorylation. Curcumol reduces the protein levels of HIF-1α and VEGF in the tumor tissues and inhibits crosstalk between STAT3 and HIF-1α pathways, thereby restricting tumor growth and progression. Evodiamine significantly diminishes MUC1-C expression, modulating tumor growth and development. Licochalcone A inhibits p65 and IKKα/β phosphorylation as well as, nuclear translocation, thus negatively regulating NF-κB and Ras/Raf/MEK signaling pathways. Erianin regulates Ras, VEGF, and HIF-1α expression to inhibit tumoral growth and progression. β-elemene suppresses the expression of p-Akt while lycopene reduces the levels of IL-4 and IL-10, with enhanced levels of antitumor T cells. Similar activation of T cells is observed with panaxadiol, which restores T-lymphocytes tumor-killing activity significantly enhancing the specific lysis of tumor cells. Both sativan and polydatin affect miRNA levels, thereby regulating the key functions within tumor cell cycle pathways to restrict their proliferation. Certain bioactive compounds, such as shikonin, also lead to the inactivation of CSN5, resulting in the degradation of PD-L1 followed by subsequent activation of tumor infiltration by T cells. A brief overview summarizing the mechanisms underlying various major natural compounds is also presented in Fig. 6.

In clinical settings, radiation therapy and immunotherapy can be combined. To effectively activate the antitumor immune response, radiotherapy can trigger the release of tumor antigens, improve the immunogenicity of cancer cells, activate immune cells, produce immune factors, and promote the presentation of tumor-related antigens [440]. Early radiation therapy has been shown to improve the antitumor immune response and restore T cell function when administered PD-1/PD-L1 mAb [440].

However, the ability of radiation to elicit a systemic tumor response with associated regression of untreated metastases outside of the radiation field has been reported. This phenomenon is known as the abscopal effect [441]. Ionizing radiation therapy is widely employed for local tumor control in both definitive and metastatic settings. The preclinical and clinical development of PD-1/PD-L1 blockage in conjunction with radiation therapy is discussed elsewhere [442].

The present work delves into the emerging field of utilizing bioactive natural compounds to modulate the PD-1/PD-L1 signaling pathway for potential cancer treatment. The review highlights the significance of this pathway in immune evasion and tumor progression while exploring the therapeutic potential of natural compounds in restoring antitumor immunity. By examining recent studies, the paper emphasizes the promising results from such interventions, thereby paving the way for novel and effective approaches in cancer therapy.

## Challenges and future directions

Despite the promising potential of targeting the PD-1/PD-L1 signaling pathway with bioactive natural compounds, several challenges and pitfalls warrant consideration. Bioavailability and proper delivery of these compounds remain hurdles, necessitating innovative formulations to ensure efficient cellular uptake. Furthermore, individual variability in response to these compounds and potential off-target effects require comprehensive clinical investigations. Additionally, the complex interplay between the immune system and tumor microenvironment demands a deeper understanding to optimize therapeutic outcomes. Nevertheless, further research is needed to fully harness the benefits of these bioactive products in the treatment of tumor and disease progression, while promoting strategic research guidelines for developing new natural product-based anticancer therapies. Our review also underscores the lack of clinical trials investigating the effects of natural compounds, providing a holistic view of the current research landscape. Despite their potential anticancer effects, these compounds face numerous challenges and limitations, such as limited permeation across the cell membrane, low solubility in aqueous media, poor bioavailability, short duration in blood circulation, rapid enzymatic degradation, and an inability to distinguish cancer cells from normal cells, which can lead to serious adverse effects and organ toxicity. Apart from these limitations, the availability of raw materials and standardization pose a prominent problem for natural products. Tedious isolation strategies alongside complex chemical structures represent some major obstacles.

Researchers are increasingly employing transcriptomics and metabolomics to elucidate the underlying mechanisms of action. These complimentary methods offer important insights into the metabolic pathways and modifications in gene expression induced by phytochemicals. The thorough examination of metabolites, small molecules involved in cellular functions, is known as metabolomics. By monitoring the concentrations of different metabolites, researchers can assess a cell’s or organism’s metabolic status. Transcriptomics focuses on studying gene expression, which involves translating genetic information into functional products such as proteins. Researchers can determine which genes are actively transcribed by examining the RNA transcripts found in a cell or tissue. Integrating transcriptomics and metabolomics allows for a more thorough understanding of the mechanisms through which phytochemicals exert their effects. Both metabolomics and transcriptomics serve as effective methods for exploring the actions of phytochemicals. These techniques can advance our knowledge of the therapeutic potential of these natural compounds and facilitate the development of innovative phytochemical-based medicines by providing insights into metabolic pathways and alterations in gene expression.

Interdisciplinary approaches that combine natural compounds with synthetic drugs or other therapeutic modalities present promising avenues for developing novel and effective treatments. By leveraging the strengths of both natural compounds and synthetic drugs, these interdisciplinary strategies can offer innovative solutions to address complex health challenges and improve patient outcomes. Key advantages of this combinational approach include amplified therapeutic effects, reduced side effects, overcoming drug resistance, targeting multiple pathways, and bioenhancing properties that improve bioavailability.

Our work underscores the need for future research to address these challenges and facilitate the successful integration of bioactive natural compounds into cancer therapy. Investigating innovative delivery systems, such as nanoparticles or liposomes, could enhance the bioavailability of these compounds and improve their therapeutic efficacy. However, it is crucial to evaluate the influence of nanomaterials on the immune function. The antigenicity of nanomaterials refers to their ability to bind to T cells or antibodies, while immunogenicity pertains to their capacity to elicit humoral or cell-mediated immune responses.

Clinical trials with rigorous patient stratification are essential for determining the safety and effectiveness of these interventions. Furthermore, a comprehensive exploration of the synergistic potential between natural compounds and existing immunotherapies could unveil novel combination approaches for enhanced antitumor responses. Concurrently, deeper mechanistic insights into the intricate interactions among the PD-1/PD-L1 pathway, immune cells, and the tumor microenvironment will inform the design of more targeted and efficient interventions. Ultimately, we hope that this work lays a foundation for future research endeavors that hold the promise to reshape cancer therapy through the utilization of bioactive natural compounds to modulate the PD-1/PD-L1 signaling pathway.

## Data Availability

Not applicable.

## Abbreviations

AFAP1-AS1: Actin filament-associated protein 1 antisense RNA1
BRCA: Breast cancer gene
BRD4: Bromodomain containing 4
CD28: Cluster of differentiation 28
EGCG: Epigallocatechin gallate
CRC: Colorectal carcinoma
CSCs: Cancer-stem-like cells
CSN5: Constitutive photomorphogenic-9 signalosome 5
CTLA-4: Cytotoxic T lymphocyte associated protein 4
EGFR: Epidermal growth factor receptor
EMT: Epithelial-mesenchymal transition
ER: Estrogen receptor
ERK: Extracellular signal-regulated kinase
FDA: Food and Drug Administration
GPX4: Glutathione peroxidase 4
GSK-3β: Glycogen synthase kinase-3β
HCC: Hepatocellular carcinoma
HDAC3: Histone deacetylase 3
HER2: Human epidermal growth factor receptor 2
Hh: Hedgehog
HIF: Hypoxia-inducible factor
HIF-1α: Hypoxia inducible factor-1α
HNSCC: Head and neck squamous cell carcinoma
HPV: Human papillomavirus
ICIs: Immune checkpoint inhibitors
ICAM-1: Intercellular adhesion molecule-1
IFN-γ: Interferon-γ
IgG1: Immunoglobulin G1
IκB: Inhibitors of nuclear factor-κB
IKK: Inhibitors of nuclear factor-κB kinase
IL: Interleukin
IL4I1: IL-4 induced gene 1
irAEs: Immune-related adverse events
IRF: Interferon regulatory factor
JAK: Janus kinase
LLC: Lewis lung carcinoma
LncRNAs: Long non-coding RNAs
MAPK: Mitogen-activated protein kinase
MCC: Merkel cell carcinoma
MDSCs: Myeloid-derived suppressor cells
MHC: Major histocompatibility complex
mAb: Monoclonal antibody
miRNAs: MicroRNA
mTOR: Mammalian target of rapamycin
MMP: Matrix metalloproteinase
NF-κB: Nuclear factor-κB
NK: Natural killer
NSCLC: Non-small cell lung cancer
PARP: Poly(ADP-ribose) polymerase
PBMCs: Peripheral blood mononuclear cells
PC: Pancreatic cancer
PDAC: Pancreatic ductal adenocarcinoma
PD-1: Programmed cell death protein 1
PD-L1: Programmed cell death ligand 1
PD-L2: Programmed cell death ligand 2
PI3K: Phosphatidylinositol 3-kinase
PR: Progesterone receptor
PTEN: Phosphatase and TENsin homolog
Sema3A: Semaphorin-3A
SNHG14: Small nucleolar RNA host gene 14
STAT: Signal transducer and activator of transcription
TCR: T cell receptor
TGF-β: Transforming growth factor-β
Th1: T helper type 1
TNBC: Triple-negative breast cancer
TNF-α: Tumor necrosis factor-α
Tregs: Regulatory T cells
UCA1: Urothelial carcinoma associated 1
VEGF-A: Vascular endothelial growth factor A
ZEB1: Zinc finger E-box binding homeobox 1
